# Temporal profiling of therapy resistance in human medulloblastoma identifies novel targetable drivers of recurrence

**DOI:** 10.1126/sciadv.abi5568

**Published:** 2021-12-08

**Authors:** David Bakhshinyan, Ashley A. Adile, Jeff Liu, William D. Gwynne, Yujin Suk, Stefan Custers, Ian Burns, Mohini Singh, Nicole McFarlane, Minomi K. Subapanditha, Maleeha A. Qazi, Parvez Vora, Michelle M. Kameda-Smith, Neil Savage, Kim L. Desmond, Nazanin Tatari, Damian Tran, Mathieu Seyfrid, Kristin Hope, Nicholas A. Bock, Chitra Venugopal, Gary D. Bader, Sheila K. Singh

**Affiliations:** 1 McMaster Stem Cell and Cancer Research Institute, McMaster University, Hamilton, ON, Canada.; 2 Department of Biochemistry and Biomedical Sciences, McMaster University, Hamilton, ON, Canada.; 3 The Donnelly Centre, University of Toronto, Toronto, ON, Canada.; 4 Department of Surgery, Faculty of Health Sciences, McMaster University, Hamilton, ON, Canada.; 5 Department of Psychology, Neuroscience and Behaviour, McMaster University, Hamilton, ON, Canada.; 6 Lunenfeld-Tanenbaum Research Institute at Mount Sinai Hospital, Toronto, ON, Canada.; 7 Princess Margaret Cancer Centre at University Health Network, Department of Molecular Genetics and Department of Computer Science, Toronto, ON, Canada.

## Abstract

Medulloblastoma (MB) remains a leading cause of cancer-related mortality among children. The paucity of MB samples collected at relapse has hindered the functional understanding of molecular mechanisms driving therapy failure. New models capable of accurately recapitulating tumor progression in response to conventional therapeutic interventions are urgently needed. In this study, we developed a therapy-adapted PDX MB model that has a distinct advantage of generating human MB recurrence. The comparative gene expression analysis of MB cells collected throughout therapy led to identification of genes specifically up-regulated after therapy, including one previously undescribed in the setting of brain tumors, bactericidal/permeability-increasing fold-containing family B member 4 (*BPIFB4*). Subsequent functional validation resulted in a markedly diminished in vitro proliferation, self-renewal, and longevity of MB cells, translating into extended survival and reduced tumor burden in vivo. Targeting endothelial nitric oxide synthase, a downstream substrate of BPIFB4, impeded growth of several patient-derived MB lines at low nanomolar concentrations.

## Teaser

Gene expression profiling of medulloblastoma cells undergoing therapy identifies BPIFB4 as a novel target for recurrent disease.

## INTRODUCTION

Originating in the cerebellar region of the brain, medulloblastoma (MB) represents the most common malignant pediatric brain tumor. Although currently established multimodal standard-of-care (SoC) therapy for MB consisting of surgical resection, cytotoxic chemotherapy, and radiation for noninfant patients has brought survival rates to 70 to 85% in standard-risk patients (*1*, *2*), the 5-year overall survival (OS) remains less than 70% for high-risk patients (*3*, *4*). Over the past decade, genomic profiling of primary MB characterized molecular heterogeneity within MB cohorts and led to subsequent stratification of MB into four consensus subgroups, each distinct in prognosis and predicted therapeutic response (*5*, *6*). Of the four subgroups, patients with group 3 MB (G3 MB) experience the highest frequency of metastatic dissemination (~45%) and the worst clinical prognosis (*7*). Tumor recurrence continues to be the most adverse event in MB pathogenesis, as only 6% of relapsed patients have OS exceeding 5 years (*8*), with standard clinical care focusing on palliation rather than therapeutic intervention. Current salvage rates of recurrent MB remain dismal at less than 10%, irrespective of the treatment modality used (*7*), while the development of novel therapeutics for these patients is further encumbered by the paucity of both human samples and mouse models able to recapitulate MB recurrence.

If MB can be contextualized as a cancer in which development has gone awry, it is a great candidate to be studied through the lens of the cancer stem cell (CSC) hypothesis. It has been suggested that CSCs are endowed with conventional chemotherapy and radiation resistance, along with tumor-initiating and metastatic properties that correlate with increased tumor recurrence and poor clinical outcome (*9*). Current anticancer therapies have a tendency to kill the bulk tumor, rather than specifically target intrinsically resistant CSCs that proliferate following completion of standard therapies. The collective role of brain tumor stem cells in tumor initiation, maintenance, and therapy evasion renders them to be a consequential biological target for therapeutic development with in vitro and in vivo stem cell models as pertinent platforms for future drug discovery.

In this study, we set out to develop a therapy-adapted patient-derived xenograft (PDX) model of MB recurrence that allows for comprehensive and dynamic profiling of MB cells at engraftment, after radiation, after chemoradiotherapy, and at relapse. The data collected from gene expression and functional profiling of human MB cells undergoing therapy have characterized the role of a longevity-associated factor, bactericidal/permeability-increasing fold-containing family B member 4 (BPIFB4), in maintaining a stem cell–like state of treatment-resistant MB cells. Reduced levels of *BPIFB4* in recurrent MB cells were sufficient to diminish their aggressiveness in vitro and in vivo. Despite the absence of modalities capable of targeting BPIFB4 itself, a small-molecule inhibitor targeting endothelial nitric oxide synthase (eNOS), a downstream substrate of BPIFB4, impeded in vitro growth of several MB patient-derived lines at low nanomolar concentrations and prolonged survival of mice xenografted with recurrent MB. The development of a model that can recapitulate both disease progression and its response to therapy not only is germane to multiple cancers where access to relapsed tissue is limited but also holds great promise to mitigate the rarity of matched primary and recurrent MB samples and guide the development of next-generation targeted therapeutic agents.

## RESULTS

### Therapy-adapted PDX model of MB recurrence

**Fig. 1.**
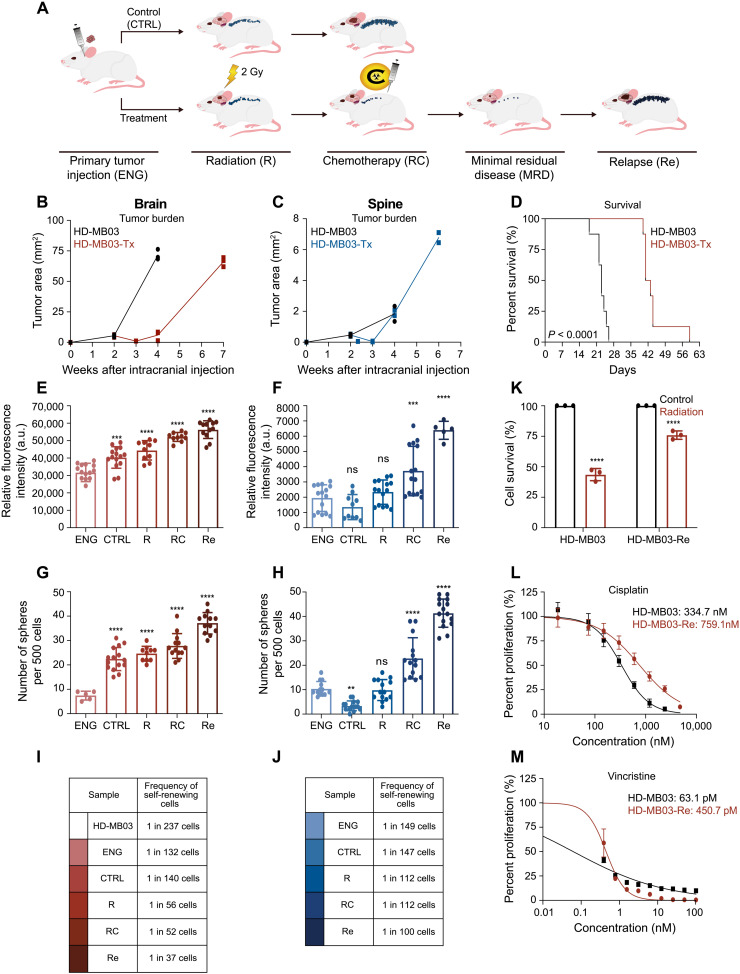
Functional profiling of HD-MB03 cells through in vivo chemoradiotherapy. (**A**) Schematic representation of the novel PDX mouse-adapted therapy model using patient-derived human G3 MB. Changes in tumor burden in (**B**) brains and (**C**) spines of xenografted mice through therapy (*n* = 3 per time point). (**D**) Kaplan-Meier curve demonstrating survival benefit of mice undergoing in vivo chemoradiotherapy (*n* = 8 per treatment arm). Proliferation assay on cells isolated from (**E**) brains and (**G**) spines of mice undergoing in vivo chemoradiotherapy (*n* = 3 per time point). a.u., arbitrary units. Changes in self-renewing potential of cells isolated from (**F**) brains and (**H**) spines of mice undergoing in vivo chemoradiotherapy (*n* = 3 per time point). ns, not significant. Fraction of self-renewing cells in cultures derived from (**I**) brains and (**J**) spines of mice undergoing in vivo chemoradiotherapy (*n* = 3 per time point). Changes in sensitivity of recurrent HD-MB03 cells to in vitro (**K**) radiation, (**L**) cisplatin, and (**M**) vincristine treatments. Bars represent the mean of at least three technical replicates. ns, not significant. *P ≤ 0.05; **P ≤ 0.001; ***P ≤ 0.0001; ****P ≤ 0.00001, unpaired *t* test or one-way analysis of variance (ANOVA) with Sidak’s method for multiple comparisons.

The clonal evolution of tumor cells in response to conventional cytotoxic therapies has become one of the biggest hurdles in designing therapies for patients with tumor recurrence (*10*, *11*). As most of the studies focus on MB at diagnosis, understanding of MB at recurrence remains limited. Our novel PDX mouse-adapted therapy model incorporates radiotherapy and chemotherapy—the two treatment modalities that exert selective pressure to approximate the clonal evolution of G3 MB in patients (Fig. 1A). Mice xenografted with representative primary human G3 MB lines, HD-MB03 and D425, were treated with craniospinal irradiation and a combination of chemotherapy drugs administered to pediatric MB patients, consisting of cisplatin, vincristine, and cyclophosphamide. Following treatment, xenografted mice demonstrated an initial response to treatment as indicated by reduced local and metastatic tumor burden (Fig. 1, B and C, and fig. S1, A to C). Although the combined therapy regimen improved the OS in both HD-MB03 and D425 cohorts (*n* = 8) by 19 and 8.5 days, respectively, all treated mice succumbed to subsequent tumor recurrence (Fig. 1D and fig. S1D). Most intriguing was the continual increase in phenotypic traits of proliferation and self-renewal through the stages of therapy in human G3 MB cells isolated from both local (brains) and metastatic (spines) compartments of the xenografted mice [Fig. 1 (E to H) and figs. S1 (E to H) and fig S2]. As the ability of a cancer cell to undergo self-renewal is one of the key pillars of the CSC hypothesis (*10*), we performed a limiting dilution assay (LDA) to identify any changes in the frequency of self-renewing cells in samples isolated from brains and spines at each stage of therapy. A major increase in incidence of self-renewing cells was observed in samples extracted from brains (HD-MB03: 1 of 132 at engraftment and 1 of 37 at relapse; Fig. 1I and fig. S1I), compared to a more modest increase in frequency of self-renewing cells isolated from spines (HD-MB03: 1 of 147 at engraftment and 1 of 100 at relapse; Fig. 1J and fig. S1J). These data suggest that self-renewal is not a phenotypic trait required by cells with the potential for leptomeningeal metastasis, further highlighting the bicompartmental nature of the disease (*12*) and the need for development of therapies specific to metastatic cells. Studies profiling CSCs have shown increased resistance to chemoradiotherapy (*13*, *14*) when compared to more differentiated tumor cells. To ensure that our model was able to select for the cells capable of evading therapy, we measured the response of G3 MB cells isolated at relapse to in vitro retreatment with radiation, cisplatin, and vincristine. In all three cases, we observed an increased tolerance of cells collected at relapse to each of the treatment modalities (Fig. 1, K to M, and fig. S1, K to M).

### Gene expression profiling identifies dynamic evolution of MB cell through therapy

**Fig. 2.**
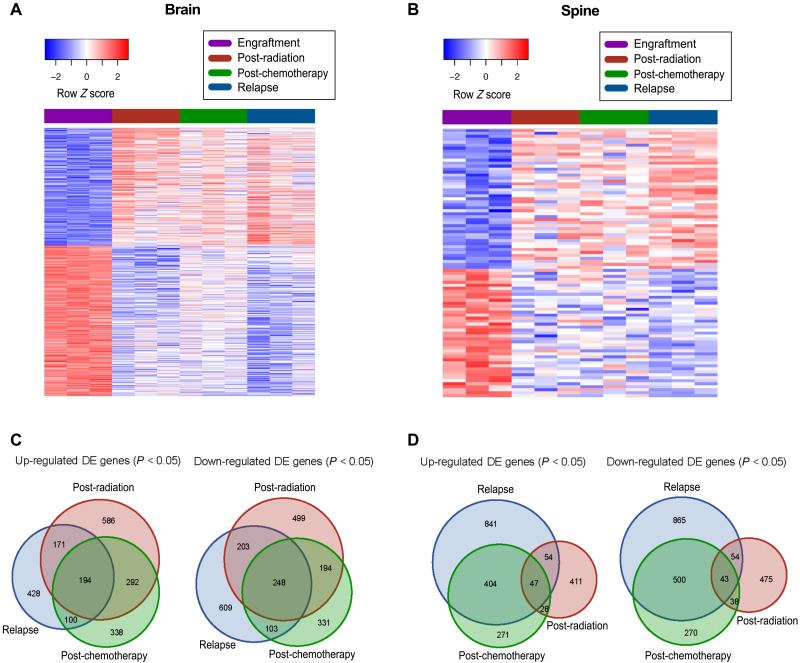
RNA-sequencing analysis of HD-MB03 cells undergoing in vivo chemoradiotherapy treatment. A heat map of differentially expressed genes in cells isolated from (**A**) brains and (**B**) spines through the course of in vivo treatment. Venn diagrams representing the number of differentially expressed (DE) genes of cells isolated from (**C**) brain and (**D**) spines at each stage of therapy, compared to the engraftment time point.

**Fig. 3.**
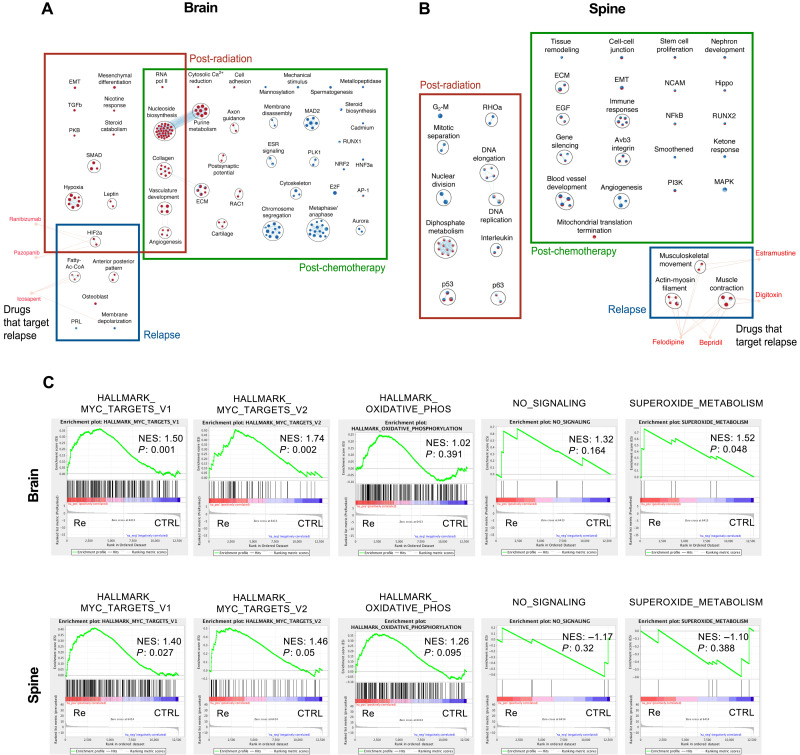
Pathway analysis of HD-MB03 cells undergoing in vivo chemoradiotherapy treatment. Differential expression profiles were used to generate pathway maps representative of significantly dysregulated pathways in (**A**) brain and (**B**) spine samples. (**C**) Comparative expression levels of Myc targets, oxidative phosphorylation, NO signaling, and superoxide metabolism between HD-MB03 cells isolated at relapse versus untreated counterpart.

The utility of our PDX mouse-adapted therapy model lies in its ability to generate a comprehensive gene expression comparison between human treatment-naïve and recurrent G3 MB to identify driver genes of therapy evasion and subsequent relapse. The initial gene expression profiling of cells isolated from brains and spines (Fig. 2, figs. S3 and S4, and table S1) and subsequent pathway analysis (Fig. 3, A and B, fig. S5, and tables S2 and S3) revealed distinct alterations in the underlying pathways driving G3 MB cell growth in response to selective pressures exerted by either radiotherapy and chemotherapy. With very few pathways overlapping between each stage of treatment, our model has captured the dynamic nature of G3 MB cells as they undergo therapy. In accordance with previously published reports (*15*), pathway analysis revealed up-regulation of *Myc* target genes in samples isolated at relapse (Fig. 3C). In addition to validating genes that have been previously described as modulators of CSCs such as proteins belonging to the family of inhibitors of DNA (ID) (fig. S6, A to D) (*16*, *17*), our differential expression analysis identified genes yet to be described in the context of cancer. Most intriguing was the observation of consistent overexpression (OE) of BPIFB4 in tumor samples collected from the brains of xenografted mice after therapy in both datasets (table S1 and fig. S4).

### Increased BPIFB4 mRNA levels correlate with aggressive MB phenotype in vitro and in vivo

**Fig. 4.**
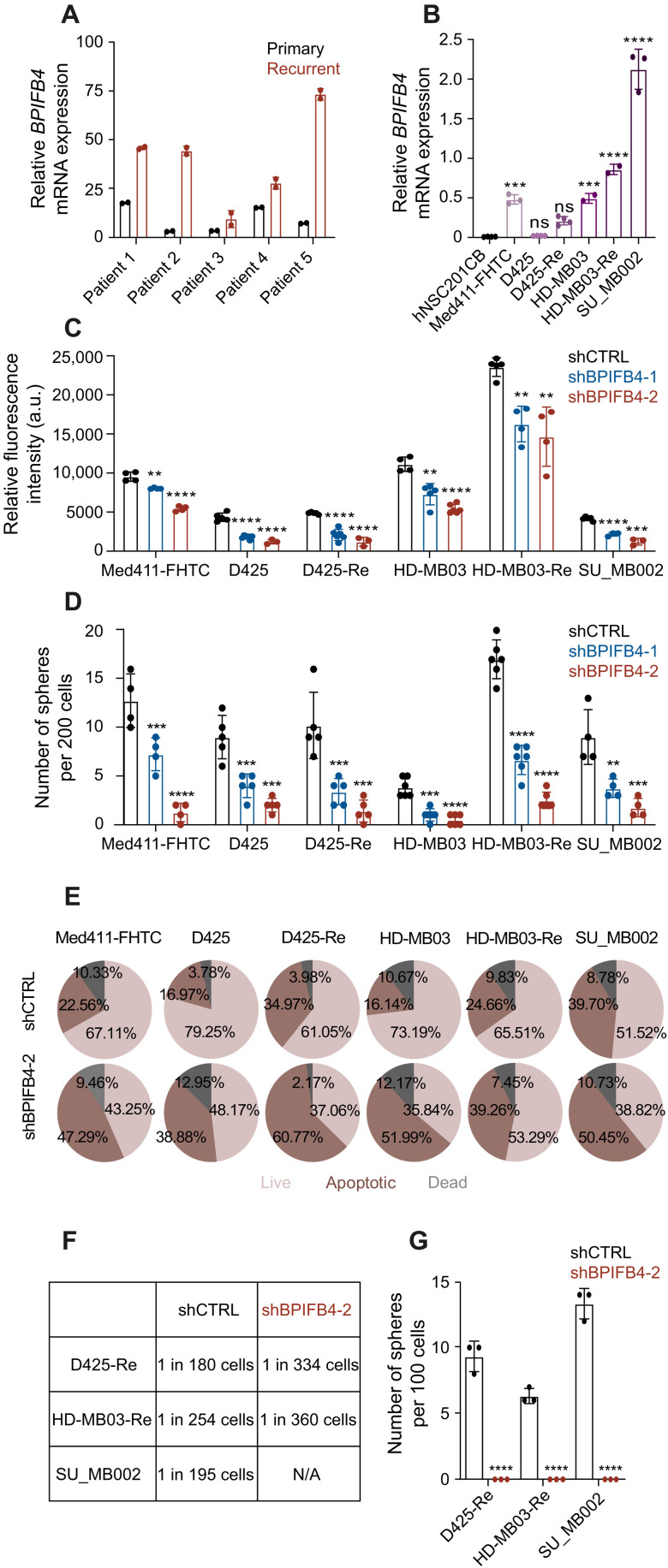
In vitro effects of BPIFB4 KD in MB cell lines. (**A**) *BPIFB4* mRNA levels in matched primary and recurrent patient samples. Samples from patients 1, 2, and 3 are representative of SHH MB, while samples from patients 4 and 5 are consistent with group 4 MB. (**B**) Relative *BPIFB4* mRNA expression levels in hNSCs and six MB cell lines. Changes in (**C**) proliferation, (**D**) self-renewal, (**E**) fraction of cells undergoing apoptosis, and (**F**) frequency of self-renewing cells in MB cell cultures after lentivector-mediated KD of BPIFB4. (**G**) Abrogation of self-renewal capacity after third in vitro passage of three recurrent MB lines with BPIFB4 KD. Bars represent the mean of at least three technical replicates. **P ≤ 0.001; ***P ≤ 0.0001; ****P ≤ 0.00001, unpaired *t* test or one-way ANOVA with Sidak’s method for multiple comparisons.

To determine the clinical significance of *BPIFB4* up-regulation at MB recurrence, we profiled *BPIFB4* mRNA expression in a collection of 19 MB samples representing the four consensus molecular subtypes (WNT: *n* = 4; SHH: *n* = 6; group 3: *n* = 3; group 4: *n* = 6; table S4 and fig. S6, E to G). Although increased *BPIFB4* expression did not associate with a specific MB subgroup either in our cohort or in the large dataset of published primary MB samples (*5*), profiling of the available five patient matched primary and recurrent samples (SHH and group 4 MB) revealed a consistent up-regulation of *BPIFB4* at relapse (Fig. 4A). To assay *BPIFB4* expression levels in G3 cell lines, we compared primary and recurrent lines and again found BPIFB4 mRNA enrichment in recurrent disease when compared to their matched treatment-naïve counterparts and healthy human neural stem cells (hNSCs) (Fig. 4B). Although these data preclude us from making any association between subgroup and *BPIFB4* expression, the increase in *BPIFB4* expression at recurrence is reproducible across multiple datasets. To investigate whether BPIFB4 may contribute to the aggressive phenotype of recurrent G3 MB, we undertook functional studies using two different short hairpin RNAs (shRNAs) targeting *BPIFB4* in both treatment-naïve and recurrent G3 MB cell lines (fig. S6I). In all six G3 MB cell lines tested—Med-411FHTC, D425, D425-Re, HD-MB03, HD-MB03-Re, and SU_MB002—we observed a marked decrease in proliferation (Fig. 4C) and self-renewal (Fig. 4D). The reduced proliferation rate observed after BPIFB4 knockdown (KD) could be attributed to an increased number of cells undergoing apoptosis, as indicated by the Annexin V assay (Fig. 4E). Furthermore, BPIFB4 KD in three recurrent G3 MB lines led to a decreased fraction of self-renewing cells (Fig. 4F) and eventual abrogation of self-renewal after three passages in vitro (Fig. 4G), further validating its role in regulating the longevity of G3 MB cells. Last, the reduction of *BPIFB4* expression in the recurrent G3 MB cells leads to a marked sensitization to in vitro chemoradiotherapy, highlighting a potentially protective role of BPIFB4 in MB cells against cytotoxic therapies (fig. S7A). The functional role of BPIFB4 in MB cells was further evaluated in three primary G3 MB lines lentivirally transduced with BPIFB4 OE vectors (D425, HD-MB03, and Med-411FHTC; fig. S7B). In all three lines, increased levels of *BPIFB4* mRNA expression led to increased proliferation and self-renewal (fig. S7, C to E) and decreased sensitivity to in vitro chemoradiotherapy (fig. S7F).

**Fig. 5.**
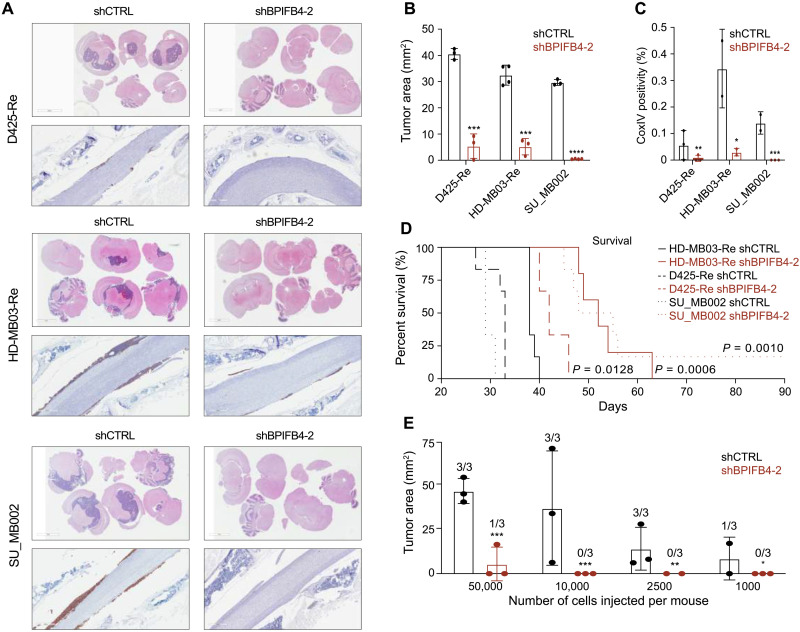
In vivo effects of BPIFB4 KD in MB cell lines. (**A**) Representative immunohistochemistry of brain and spine sections stained with hematoxylin and eosin and human-CoxIV staining, respectively. Quantified tumor burden in (**B**) brains and (**C**) spines of mice xenografted with recurrent MB cells transduced with control or BPIFB4 KD lentivectors as indicated by measured tumor area or positivity of CoxIV staining, respectively. (**D**) Kaplan-Meier curves demonstrating extended mouse survival in MB cells with BPIFB4 KD (*n* = 6 per cohort). (**E**) Quantitative tumor burden of mice xenografted with decreasing cell numbers of shCTRL- or shBPIFB4-transduced recurrent G3 MB cells. Bars represent mean of at least three technical replicates. *P ≤ 0.05; **P ≤ 0.001; ***P ≤ 0.0001; ****P ≤ 0.00001, unpaired *t* test.

We next undertook intracranial xenotransplantation of recurrent G3 MB cells that were stably transduced with BPIFB4 KD lentivector and evaluated its effects on tumorigenicity. In all three recurrent MB lines (D425-Re, HD-MB03-Re, and SU_MB002), reduced *BPIFB4* expression translated into a reduced tumor burden in both brains and spines (Fig. 5, A to C) and prolonged survival (Fig. 5D). Subsequent in vivo LDA further corroborated observation of decreased tumorigenic potential of cells after BPIFB4 KD. In contrast to shCTRL-transduced cells that formed tumors even at 1000 cells per mouse, only one mouse xenografted with 50,000 shBPIFB4-transduced cells formed an observable tumor (Fig. 5E).

### Targeting downstream substrate of BPIFB4 provides a previously unidentified therapeutic option for recurrent MB

**Fig. 6.**
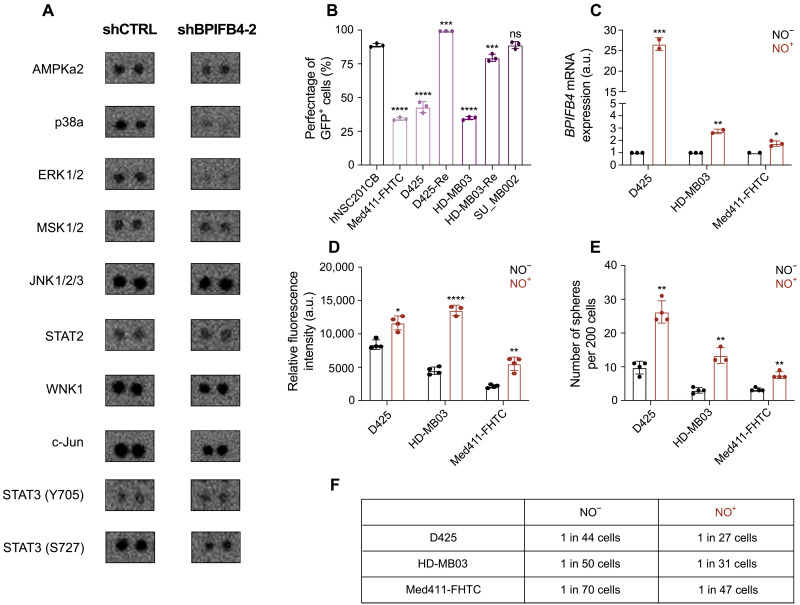
NO-producing fraction of primary G3 MB exhibits elevated BPIFB4 levels and mimics functional phenotype observed after chemoradiotherapy. (**A**) Decreased phosphorylation levels of select proteins in response to BPIFB4 KD in HD-MB03-Re. (**B**) Increased levels of NO in recurrent MB cells as indicated by an increased percentage of GFP^+^ (green fluorescent protein–positive) cells in response to treatment with NO probe. (**C**) Elevated BPIFB4 mRNA levels in NO-producing fraction of primary G3 MB cells. Changes in (**D**) proliferation, (**E**) self-renewal, and (**F**) frequency of self-renewing cells in NO-expressing fraction of primary MB cell. Bars represent the mean of at least three technical replicates. *P ≤ 0.05; **P ≤ 0.001; ***P ≤ 0.0001; ****P ≤ 0.00001, unpaired *t* test or one-way ANOVA with Sidak’s method for multiple comparisons.

**Fig. 7.**
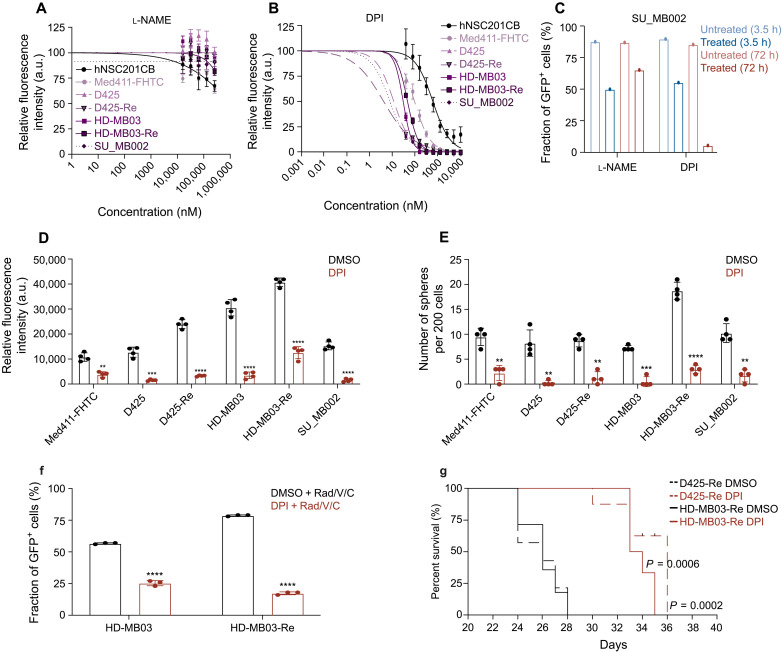
Irreversible small-molecule targeting of eNOS reduces MB proliferation in vitro and in vivo. IC_50_ curves of (**A**) l-NAME and (**B**) DPI in hNSCs and five MB lines. (**C**) In vitro evaluation of short- and long-term changes in NO levels after treatment with a reversible inhibitor, l-NAME, and an irreversible inhibitor, DPI. Changes in (**D**) proliferation and (**E**) self-renewal of MB cells treated with DPI at concentrations equivalent to IC_80_ for the respective cell line. (**F**) Enhanced efficacy of in vitro chemoradiotherapy after treatment of MB cells with DPI. (**G**) Kaplan-Meier curves demonstrating extended mouse survival in MB cells with BPIFB4 KD (*n* = 6 per cohort). Bars represent the mean of at least three technical replicates. **P ≤ 0.001; ***P ≤ 0.0001; ****P ≤ 0.00001, unpaired *t* test or one-way ANOVA with Sidak’s method for multiple comparisons.

The limited body of literature on BPIFB4 suggested its role in the activation of eNOS (NOS3) and subsequent production of NO (*18*). To gain mechanistic insights into downstream targeting of BPIFB4, we used the Human Phospho-Kinase Array to assess changes in phosphorylation status of a number of important signaling modulators in HD-MB03-Re with BPIFB4 KD. The top four most affected proteins were ERK1/2 (extracellular signal–regulated kinase 1/2), p38a, c-Jun, mTOR (mammalian target of rapamycin), and AMPKa2, all of which have been implicated in regulating the NO production pathway, providing further evidence of BPIFB4 involvement in this pathway (Fig. 6A and figs. S8 and S9) (*19***–***22*). Profiling cells with the NO reporter probe, DAF-FM, we observed increased levels of NO in recurrent G3 MB cells when compared to their treatment-naïve counterparts (Fig. 6B). Although hNSCs were also observed to have high levels of endogenous NO, its production is distinctively driven by neuronal NOS (nNOS; NOS1) (*23*). Building on the observed increase in proliferation and self-renewal in primary G3 MB cells with exogenous OE of BPIFB4, we set out to functionally compare the NO-positive and NO-negative fractions of primary MB cells. MB cells producing NO had significantly higher *BPIFB4* expression (Fig. 6C) levels in addition to markedly increased proliferation and self-renewal potential (Fig. 6, D to F). Unlike BPIFB4, eNOS can be targeted using small-molecule inhibitors. Notably, a reversible eNOS inhibitor, N^G^-nitro-l-arginine methyl ester (l-NAME), was ineffective in reducing growth of G3 MB cell lines (Fig. 7A), while an irreversible inhibitor, diphenyleneiodonium chloride (DPI), was potent at low nanomolar concentrations (Fig. 7B). When compared to recurrent MB samples, the inhibitory concentration of DPI was found to be 3- to 10-fold higher in hNSCs, providing a therapeutic window for treating patients at relapse. A comparison of short- and long-term effects of l-NAME and DPI on NO levels in recurrent G3 MB cells revealed that, unlike l-NAME, DPI was able to induce and subsequently sustain the inhibition of NO production (Fig. 7C), resulting in a phenotype closely resembling one induced by the KD of BPIFB4. Functionally, DPI-directed irreversible eNOS inhibition caused a reduction in G3 MB cell proliferation (Fig. 7D) and self-renewal (Fig. 7E), which were comparable to those observed in recurrent G3 MB cells after BPIFB4 KD. As prolonged NO exposure has been linked to chemoradiotherapy resistance (*24*), we tested the effects of DPI treatment on sensitization to chemoradiotherapy. DPI-pretreated HD-MB03 and HD-MB03-Re showed a greater response to in vitro irradiation combined with cisplatin and vincristine, when compared to dimethyl sulfoxide (DMSO)–pretreated counterparts (Fig. 7F). Furthermore, the sensitivity to DPI treatment was higher in the BPIFB4 KD setting, suggesting an additive effect (fig. S10G) of direct eNOS targeting and reduction in BPIFB4-driven eNOS activation. Our initial in vitro findings of eNOS targeting were further validated by in vivo administration of DPI to mice xenografted with recurrent MB, which resulted in prolonged survival (Fig. 7G).

## DISCUSSION

Current designs of clinical trials for patients with recurrent MB rely on genomic profiling of the primary, treatment-naïve tumor and are not specific to the highly divergent tumor presented at recurrence (*7*). The experimental approach taken in our work aims to mitigate the paucity of matched primary and recurrent samples and allow profiling of human G3 MB as it evolves through chemoradiotherapy. Functionally, cells undergoing therapy appear to have an increased propensity to self-renew in response to selective pressures exerted by chemoradiotherapy. This work demonstrates that inhibition of *BPIFB4* expression significantly impairs G3 MB stem cell self-renewal, thus implicating BPIFB4 as a driver of self-renewal of treatment-resistant G3 MB stem cells, leading to MB recurrence. While primary MBs have a relatively low frequency of somatic gene mutations, several groups have reported an increase in somatic mutational burden including mutations in *TP53* and aberrant p53-Myc interactions in human MB samples collected after chemoradiotherapy (*11*, *15*, *25*). Thus, further mechanistic studies are warranted to better understand the link between genetic changes and the transcriptomic dysregulation of *BPIFB4* in the context of recurrent G3 MB. Although BPIFB4 cannot be directly targeted with a known small-molecule inhibitor, one of the few characterized downstream effectors of *BPIFB4* is known to be eNOS (NO), which can be targeted. In a study by Villa *et al.*, the authors have put forward a potential mechanism of how WT (wild-type)-BPIFB4 and the longevity-associated variant (LAV-BPIFB4) modulate eNOS activity in endothelial cells. The initial phosphorylation of BPIFB4 on Ser^75^ by PERK facilitates BPIFB4 binding to 14-3-3. The BPIFB4/14-3-3 complex is then brought into proximity to eNOS via heat shock protein 90 (HSP90), resulting in activating phosphorylation of eNOS at S1177 and subsequent increase in NO production (*18*). Whether this is the exact mechanism of action in G3 MB cells remains to be elucidated. Our efforts to gain insights into the role of BPIFB4 in G3 MB cells that have been recovered after chemoradiotherapy have demonstrated a down-regulation in phosphorylation levels in several pathways implicated in MB pathogenesis. Several of the modulated kinases in response to BPIFB4 KD, ERK1/2, p38a, c-Jun, mTOR, and AMPKa2 have been previously linked to eNOS activation, providing a potentially convergent target for therapeutic intervention. While phosphokinase array showed no reduction in S1177 phosphorylation in response to BPIFB4 KD, there remain several other eNOS activating phosphorylation sites to be investigated further including Y81, S615, and S633 (*26*). A key observation emerging from profiling gene expression of G3 MB cells as they undergo therapy is up-regulation of genes contributing to enhanced cellular levels of NO. Since its identification in 1987 (*27*, *28*), NO has emerged as a molecule of interest in cancer. Currently, the exact role of NO in cancer cells remains unclear, as there are studies suggestive of both its tumoricidal (*29*, *30*) and tumor-promoting effects (*31***–***33*). NO is endogenously synthesized by a family of NOS enzymes: NOS1 (nNOS), NOS2 [inducible NOS (iNOS)], and NOS3 (eNOS). Unlike the iNOS, *nNOS* and *eNOS* are constituently expressed in neurons and endothelial cells, respectively (*34*). eNOS-driven NO production largely depends on the calcium concentration in the tissue, while the concentration of NO dictates its downstream effects (*35*, *36*). In MB, altered cytosolic Ca^2+^ activity has been previously correlated with therapy resistance (*37*). While in some cancers increased NO levels can contribute to regulation of cell cycle, angiogenesis, invasion, and metastasis (*38*), it can also lead to the induction of proapoptotic signaling, up-regulation of *TP53*, and suppression of DNA synthesis. The dichotomous nature of NO in cancer remains a hurdle in developing therapies exploiting NO signaling cascade, and thus, further tumor type–specific studies are required. The exact role of NO in G3 MB initiation, progression, metastasis, and relapse remains unknown and requires further investigation with the new generation of genomic tools.

Our studies targeting eNOS with a selective small-molecule inhibitor led to increased survival of mice xenografted with recurrent G3 MB. This finding, in combination with functional effects of BPIFB4 KD and up-regulation of BPIFB4 mRNA levels in recurrent patient samples profiled, may suggest the potential for a unique therapeutic option for patients with G3 MB relapse, irrespective of molecular stratification at diagnosis. We then examined our data for insights to inform clinical trial design, considering whether treatment of G3 MB with eNOS inhibitors would yield greater benefit delivered up-front and concurrent with SoC or subsequent to standard therapy upon diagnosis of recurrence. In our model, we initially observed low expression levels of *BPIFB4* at engraftment, with progressive enrichment of *BPIFB4* levels through in vivo chemoradiotherapy. Furthermore, elevated BPIFB4 mRNA levels were found in primary G3 MB cells sorted on the basis of NO production. Subsequent comparison of cell fractions sorted on the basis of NO levels exposed the similarities in functional phenotypes between NO-positive primary G3 MB cells and those isolated after chemoradiotherapy with increased *BPIFB4* expression. This suggests that BPIFB4 marks a population of brain tumor–initiating cells endowed with the ability to escape standard therapy to drive recurrence, as described in the classical CSC hypothesis. As *BPIFB4* expression does not seem to arise as a new subclonal event after therapy (which would necessitate treatment only at recurrence) but rather represents a low-frequency clonal event in a primary tumor–initiating cell that is selected for survival through therapy, it should thus be targeted up-front. Further lineage-tracing experiments on BPIFB4-expressing cells are warranted to determine the correct timing of eNOS inhibitor delivery within current MB treatment regimens. As several ongoing phase 1b/2 clinical trials testing the efficacy of competitive NOS inhibitors in combination with chemotherapy drugs for aggressive solid tumors (NCT02834403 and NCT03236935), our study provides strong evidence for the potential of eNOS inhibitors as a previously undescribed treatment paradigm for patients with recurrent G3 MB. Further preclinical studies incorporating specific, irreversible eNOS inhibitors in combination with SoC are warranted to better discern the therapeutic application and rational design of early clinical studies.

Insights into molecular drivers of MB stem cell self-renewal will continue to guide identification of targeted therapies allowing for selective killing of MB stem cells while sparing the normal cells of the developing cerebellum. Our PDX therapy–adapted model permitted a genomic characterization of human tumor cells as they evolve through therapy, which led to the identification of genes, that specifically drive G3 MB recurrence. This model could promise great utility if adapted for other cancers in which human tissue is not easily recovered at relapse, and the unique genomic landscape at recurrence remains poorly defined. Similar to other cancers, it is unlikely that targeting one driver gene in a heterogeneous tumor will result in a durable treatment effect; thus, it is important to investigate synergistic effects between multiple inhibitors to achieve a global and sustained response.

## METHODS

### Human G3 MB cell cultures

MB cell lines were cultured in NeuroCult Complete (NCC) medium: NeuroCult NS-A Basal Medium supplemented with 50 ml of NeuroCult Supplement (STEMCELL Technologies), epidermal growth factor (20 ng/ml), fibroblast growth factor (10 ng/ml), 0.1% heparin, and 1% penicillin-streptomycin for a minimum of 48 hours before experiments. SU_MB002 was derived at recurrence from a patient who received only cyclophosphamide and displayed expression markers of G3 MB (*39*). HD-MB03 was isolated from a patient with metastasized G3 MB (*40*). Med-411FHTC P2 was purchased from Brain Tumor Resource Laboratory. D425-Med (D425) (*41*) was propagated in Dulbecco’s modified Eagle’s medium (DMEM) high glucose (Life Technologies) supplemented with 1% penicillin-streptomycin and 20% fetal bovine serum. hNSCs were isolated using a previously described protocol and cultured in NCC medium.

### Intracranial xenografting of MB and in vivo treatment protocol

All in vivo studies were performed according to McMaster University Animal Research Ethics Board (AREB)–approved protocols. Intracranial injections were performed as previously described (*42*). Cell numbers sufficient to generate a measurable tumor burden were previously determined and are as follows: D425, 1 × 10^4^; HD-MB03, 1 × 10^6^; and SU_MB002, 5 × 10^5^. Nonobese diabetic (NOD) severe combined immunodeficient (SCID) mice were anesthetized using isoflurane gas (5% induction and 2.5% maintenance), and 10 μl of cells was injected into the frontal lobe using a 50-μl Hamilton syringe in a nonrandomized, nonblinded fashion. The mice designated to receive treatment were subjected to 2 Gy of craniospinal irradiation using Gammacell 3000 irradiator 14 days after engraftment. The mice were irradiated in the specially designed cerrobend shield that allowed negation of full-body radiation effects while exposing the cranium and upper portion of the spine to the full dose of radiation. Following radiation, mice were allowed to recover for a week before treatment with cisplatin (2.5 mg/kg), vincristine (0.4 mg/kg), and, a day later, cyclophosphamide (75 mg/kg). Mice were closely monitored, and changes in tumor burden and survival were recorded.

For the in vivo KD studies, mice were intracranially xenografted with 1.5 × 10^4^ cells transduced with either shCTRL or shBPIFB4-2 lentiviral vectors. The number of mice allocated per experimental group was determined using the following formula: N = 1 + 2*C*(s/d)^2^, where *N* is the number of mice per treatment arm, *C* = 7.85 (significance level of 5% with a power of 80%), *s* is the SD, and *d* is the difference to be detected.

To assess tumor volume, mice were sacrificed when the control group reached endpoint. For survival studies, treated or control mice were sacrificed when they reached endpoint. Upon reaching the endpoint, brains and spines were harvested, formalin-fixed, and paraffin-embedded for hematoxylin and eosin (H&E), CoxIV, and Ki-67 staining. Images were captured using the Aperio Slide Scanner and analyzed using ImageScope v11.1.2.760 software (Aperio). CoxIV and Ki-67 positivity was determined by Positive Pixel Algorithm v9 using ImageScope v11.1.2.760 software (Aperio).

### Magnetic resonance imaging

Three-dimensional (3D) magnetic resonance imaging was performed on a 7T Bruker Ascend 300WB vertical bore with the MicWB40 probe (Bruker BioSpin). The image volume was acquired with 150-μm isotropic voxels, with a field of view of 25 mm × 25 mm × 20 mm. Magnetization transfer weighted images were acquired according to the protocol by Watanabe *et al.* (*43*). The saturation pulse was applied once per repetition time (TR), with a Gaussian shape, pulse width 12 ms, nominal flip angle 523° (maximum pulse amplitude, 6.8 μT), and offset frequency 2500 Hz. Image acquisition was performed with a spoiled gradient echo with TR of 23 ms, TE (time to echo) of 3 ms, and excitation angle of 5°. The scan time for a single 3D image was 8 min and 28 s. Eight averages were performed for a total imaging time of 1 hour, 7 min, and 11 s. Tumor-bearing animals were imaged at multiple time points after injection (in increments of weeks) to establish engraftment and progression after treatment. For magnetic resonance imaging, animals were induced with 5% isoflurane in O_2_ (1 liter/min) in an induction chamber and maintained during imaging with 1 to 2% isoflurane delivered via a nose cone. Anesthetized mice were placed on a custom plastic sled, secured with foam and Transpore tape (3M), and loaded head up into the vertical wide-bore spectrometer. Rectal temperature and breath rate were monitored with a Biopac acquisition system (MP36E-CE, Biopac Systems Inc., RRID:SCR_014829). The temperature of water circulating through the gradient was set to 35°C at the cooling unit (BCU20, Bruker BioSpin), which reliably maintained animal internal temperature at 37°C. Isoflurane levels were manually adjusted to maintain stable respiration rate of 70 breaths/min.

### Flow cytometric analysis

To isolate human MB cells from mouse tissue, xenograft samples were cultured as previously described (*44*) and sorted on the basis of human TRA-1-85 marker using a MoFlo XDP cell sorter (Beckman Coulter). MB tumor spheres were mechanically dissociated and resuspended in phosphate-buffered saline + 2 mM EDTA (Invitrogen) before staining with human anti–TRA-1-85 (1:10; Miltenyi Biotec, REA476) antibody or matched isotype control. Cells were incubated for 15 min at room temperature and run on a MoFlo XDP cell sorter. Dead cells were excluded on the basis of the viability dye 7AAD (7-aminoactinomycin D) (1:10; Beckman Coulter), and compensation values were determined using IgG CompBeads (BD Biosciences). Regions of TRA-1-85 positivity and negativity were established on the basis of isotype control.

For assessing changes in cell cycle, the propidium iodide–based Coulter DNA Prep Kit (Beckman Coulter, #6607055) was used. No deviation from the manufacturer’s instructions was introduced. To assess the extent of apoptosis, MB cells were dissociated, and single-cell suspension was resuspended in 100 μl of Annexin V Binding Buffer (BioLegend), with 2 μl of 7AAD and 1 μl of anti–Annexin V antibody (1:100; Life Technologies, #A23204). Samples were incubated at room temperature for 15 min and spun at 1100 rpm for 3 min. Cells were resuspended in 300 μl of Annexin V Binding Buffer and profiled using a MoFlo XDP cell sorter.

### Cell proliferation assay

Single-cell suspension of MB cells was sorted into a 96-well plate at a density of 1000 cells per well in 200 μl of NCC with six technical replicates per sample. After 4 days, 20 μl of PrestoBlue Cell Viability Reagent (Life Technologies) was added to each well approximately 4 hours before the readout time point. Fluorescence was measured using the FLUOstar Omega Microplate Reader (BMG Labtech) at excitation and emission wavelengths of 540 to 570 nm, respectively. Readings were analyzed using Omega software.

### Self-renewal and in vitro LDA

Single-cell suspension of MB cells was sorted into a 96-well plate at a density of 200 cells per well (500 cells per well in the case of KD experiments) in 200 μl of NCC with six technical replicates per sample. Self-renewal was evaluated by counting the number of spheres (clusters equal to or more than seven cells) formed in each well after 4 days. For in vitro limiting dilution analysis, viable cells were sorted in quadruplicates into a 96-well plate using MoFlo XDP at cell densities ranging from 1000 cells per well to 1 cell per well in 200 μl of NCC. The number of wells without any spheres or colonies after 4 days was scored, and the fraction of negative wells was plotted against the number of cells per well. The number of cells corresponding to the fraction of negative wells equal to 0.37 is the dilution with one self-renewing unit (*45*).

### In vitro dose-response curves of cisplatin, vincristine, DPI, and l-NAME

Cells (1 × 10^3^) were plated in a 96-well plate in quadruplicates at a volume of 200 μl per well with twofold dilutions of cisplatin or DPI from concentrations of 20 μM to 39 nM. In the case of vincristine, the concentrations tested ranged from 200 nM to 39pM and 500 μM to 31.25 μM, respectively. Highest volume of DMSO was used as negative control. After 72 hours, proliferation was measured as described in the “Cell proliferation assay” section, and IC_50_ (median inhibitory concentration) values were determined by plotting percent cell viability versus log_10_-transformed concentration of inhibitors. Throughout the manuscript, IC_50_ value refers to the concentration of drug that was effective in reducing viability of cell culture by 50%. IC_80_ values were calculated using the following formula: IC_(*F*)_ *=* [(100 − *F*)/*F*]^1/HS^ × IC_50_ (where *F* is the percent reduction of proliferation and HS is the Hill slope).

### In vitro irradiation of MB cells

Cells were plated at a density of 2.5 × 10^5^ cells per well into a six-well tissue culture–treated plate in triplicates and were irradiated with a single dose of 2 Gy using Faxitron RX-650. After 72 hours, cell viability was assessed by mixing 10 μl of cell suspension with 10 μl of 0.4% Trypan Blue solution (Life Technologies), and the cell counts generated by the Countess II FL Automated Cell Counter (Life Technologies) were plotted.

### RNA-sequencing analysis

Gene expression data were obtained from RNA-sequenced samples as raw counts. The count data were then normalized using edgeR with CPM (counts per million) and filtered by TMM (trimmed mean of *M* values) method. TMM kept the genes with CPM greater than or equal to 2 in at least three samples as determined by comparing sample densities. Filtered CPM data were transformed by log_2_ transformation and subjected to batch correction using the package “Removing Unwanted Variation (RUV).” We used the bottom 75% and low-expressing genes for calculating correction factors by the RUV algorithm. The batch-corrected values were used for detecting differentially expressed (DE) genes by generalized linear model likelihood ratio test (glmLRT) in edgeR comparing each treatment groups to control and engraft samples.

### Pathway analysis

Rank files were generated from the *P* values and fold change in the comparisons, and pathway analysis (GSEA, Broad Institute) was performed. Significant pathways were visualized in Cytoscape (v3.6.1) using the Enrichment Map App (v3.1.0) with *P* < 0.001, FDR *q* < 0.1, and Jaccard > 0.25 for shared genes. Pathway clusters were organized and labeled by AutoAnnotate App (v1.2) in Cytoscape.

### Microarray analysis

The raw data files were combined and processed by the BioConductor package “lumi.” In short, expression data were first normalized by the quantile method and then filtered by detection FDR values. Only probes with detection FDR value < 0.05 in at least two samples were included in the analysis. In the case where multiple probes were designed for one gene (duplicates), only the probe with the highest SD was chosen. Out of 47,323 probes on the Illumina HT-12 microarray representing 22,864 unique genes, 15,281 probes/genes passed the FDR (False discovery rate) filter and duplicate removal. After quantile normalization, multidimensional scaling (MDS) plots were used to assess the difference between treatment groups in Brain and Spine. The normalized and log_2_-transformed intensity values of microarray data were used to calculate differential expression by the BioConductor package “limma.” Bayesian moderated *t*-statistics tests (ModT-test) were performed to determine DE genes, and the *T* values from ModT-tests were used as ranking scores to generate Rank files for gene set enrichment analysis (GSEA).

### Patient data

Human MB samples and clinical data were obtained from consenting patients, as approved by the Research Ethics Board at Hamilton Health Sciences. To identify the molecular subtype of each sample, RNA from patient tumor samples was isolated using the Total RNA Isolation Kit (Norgen) and submitted for NanoString nCounter profiling at Farncombe Metagenomics Facility (McMaster University). The custom CodeSet was designed using previously characterized genes for each of the core MB subgroups: WNT—*WIF1*, *TNC*, *GAD1*, *DKK2*, and *EMX2*; SHH—*PDLIM3*, *EYA1*, *HHIP*, *ATOH1*, and *SFRP1*; group 3—*IMPG2*, *GABRA5*, *EGFL11*, *NRL*, and *MAB21L2*; group 4—*KCNA1*, *EOMES2*, *KHDRBS2*, *RBM24*, *UNC5D*, and *OAS1*; and six housekeeping genes—*ACTB*, *TBP*, *LDHA*, *POLR2A*, *GAPDH*, and *HPRT1*. The subgroup assignment was determined as previously described (*46*).

### Reverse transcription quantitative polymerase chain reaction

Cells (2.5 × 10^5^) were collected and total RNA was extracted using the Total RNA Isolation Kit (Norgen). Complimentary DNA (cDNA) was synthesized using qScript cDNA SuperMix (Bio- Rad) and the C1000 Thermo Cycler (Bio-Rad) with the following cycle settings: 4 min at 25°C, 30 min at 42°C, 5 min at 85°C, hold at 4°C. Reverse transcription quantitative polymerase chain reaction (RT-qPCR) was performed using Perfecta SYBR Green (Quanta Biosciences) and a CFX96 instrument (Bio-Rad). CFX Manager 3.0 software was used for quantification of gene expression and was normalized to 28*S* ribosomal RNA (rRNA). The following primers were used to measure mRNA levels of *BPIFB4* (forward: 5′-AGATCCTTGAGTCCGAGGGAA-3′; revers: 5′-TGCGAGGATGCCATCAGC-3′), *ID1* (forward: 5′-AATCATGAAAGTCGCCAGTG-3′; reverse: 5′-ATGTCGTAGAGCAGCACGTTT-3′), *ID2* (forward: 5′-ATGAAAGCCTTCAGTCCCGT-3′; reverse: 5′-TTCCATCTTGCTCACCTTCTT-3′), *ID3* (forward: 5′-TCATCTCCAACGACAAAAGG-3′; reverse: 5′-ACCAGGTTTAGTCTCCAGGAA-3′), *28SrRNA* (forward: 5′-AAGCAGGAGGTGTCAGAAA-3′; reverse: 5′-AAAACTAACCTGTCTCACG-3′), and β-tubulin (forward: 5′-CCAACCGCGAGAAGATGACCCAGATCA-3′; reverse: 5′-GTGAGGATCTTCATGAGGTAGTCAGTC-3′).

### Lentiviral KD and OE studies

pGFP-C-shLenti vectors expressing shRNA targeting human BPIFB4 (shBPIFB4-1: 5′-TTATCCTCGGCTGGTCATTGAGCGATGTG-3′; shBPIFB4-2: 5′-ACAGTGGCTATCGCAGTGCCGAGAATGCA-3′) and the control vector (shCTRL: 5′-ATCAGTTGCTCAGATACTCAGC-3′) were purchased from OriGene (#TL305949 and #TR30023). OE BPIFB4 constructs were purchased from GeneCopoeia (EX-Y2680-LV122). Lentiviral pLKO.1 vectors expressing shRNA targeting human ID1 (shID1.1: 5′-CGGCTGTTACTCACGCCTCAA-3′; shID1.2: 5′-GCAGGTAAACGTGCTGCTCTA-3′), ID2 (shID2.1: 5′-GAGCCTGCTATACAACATGAA-3′), ID3 (shID3.1: 5′-CATCGACTACATTCTCGACCT-3′; shID3.2: 5′-GCCCACTTGACTTCACCAAAT-3′), and the control vector (shGFP: 5′-ACAACAGCCACAACGTCTATA-3′) were gifts from J. Moffat. Stable cell lines with KD or OE were generated by transduction and subsequent selection with puromycin. The extent of KD or OE was validated by RT-qPCR as previously described.

### Human phosphokinase array

The assay was performed in accordance with the manufacturer’s specifications and guidelines (R&D Systems, #ARY003B). Briefly, HD-MB03-Re cells were lentivirally transduced with shCTRL or shBPIFB4-2 vectors as per established protocols. Following selection and validation of KD, 3 × 10^6^ cells per sample were collected, lysed, and processed in accordance with the provided protocol.

### In vivo treatment of mice with DPI

Mice xenografted with 1.0 × 10^4^ D425-Re and HD-MB03-Re cells were treated with two doses per week with DPI (100 μg/kg) for 2 weeks. The number of mice allocated per experimental group was determined using the following formula: N = 1 + 2*C*(s/d)^2^, where *N* is the number of mice per treatment arm, *C* = 7.85 (significance level of 5% with a power of 80%), *s* is the SD, and *d* is the difference to be detected.

### Measuring levels of NO

Cellular levels of NO were measured by addition of 1.5 μM DAF-FM diacetate to cell cultures as previously described (*47*). The levels of GFP (green fluorescence protein) fluorescence were evaluated 1 hour after treatment with DAF-FM, and no exogenous l-arginine was added throughout the assay. Samples designated as control were treated with equal volume of DMSO.

### Statistical analysis

At least three technical or experimental replicates from each experiment were compiled. Data represent mean ± SD, with *n* values listed in figure legends. GraphPad Prism was used to plot all bar graphs and statistical analyses including Student’s *t* test or two-way analysis of variance (ANOVA) (*P* < 0.05 was considered significant). All Kaplan-Meier survival plots were plotted with GraphPad Prism, and log-rank (Mantel-Cox) test was performed for comparison of median survival (*P* < 0.05 was considered significant). For in silico analyses, all associated statistical tests were performed in R using the coxPH package.

## Notes

View/request a protocol for this paper from *Bio-protocol*.
