# Supplementary material for: Temporal profiling of therapy resistance in human medulloblastoma identifies novel targetable drivers of recurrence
Source: Sci Adv. 2021 Dec 8;7(50):eabi5568. doi: 10.1126/sciadv.abi5568 (PMC8654291; doi:10.1126/sciadv.abi5568)
Supplement: Supplementary file 1 — Figs. S1 to S10 Tables S1 to S4 [file sciadv.abi5568_sm.pdf]

Science Advances
AAAS

Supplementary Materials for

Temporal profiling of therapy resistance in human medulloblastoma identifies novel targetable drivers of recurrence

David Bakhshinyan, Ashley A. Adile, Jeff Liu, William D. Gwynne, Yujin Suk, Stefan Custers, Ian Burns, Mohini Singh, Nicole McFarlane, Minomi K. Subapanditha, Maleeha A. Qazi, Parvez Vora, Michelle M. Kameda-Smith, Neil Savage, Kim L. Desmond, Nazanin Tatari, Damian Tran, Mathieu Seyfrid, Kristin Hope, Nicholas A. Bock, Chitra Venugopal, Gary D. Bader, Sheila K. Singh*

*Corresponding author. Email: ssingh@mcmaster.ca

Published 8 December 2021, *Sci. Adv.* **7**, eabi5568 (2021)
DOI: 10.1126/sciadv.abi5568

This PDF file includes:

Figs. S1 to S10
Tables S1 to S4

Supplementary Figure 1

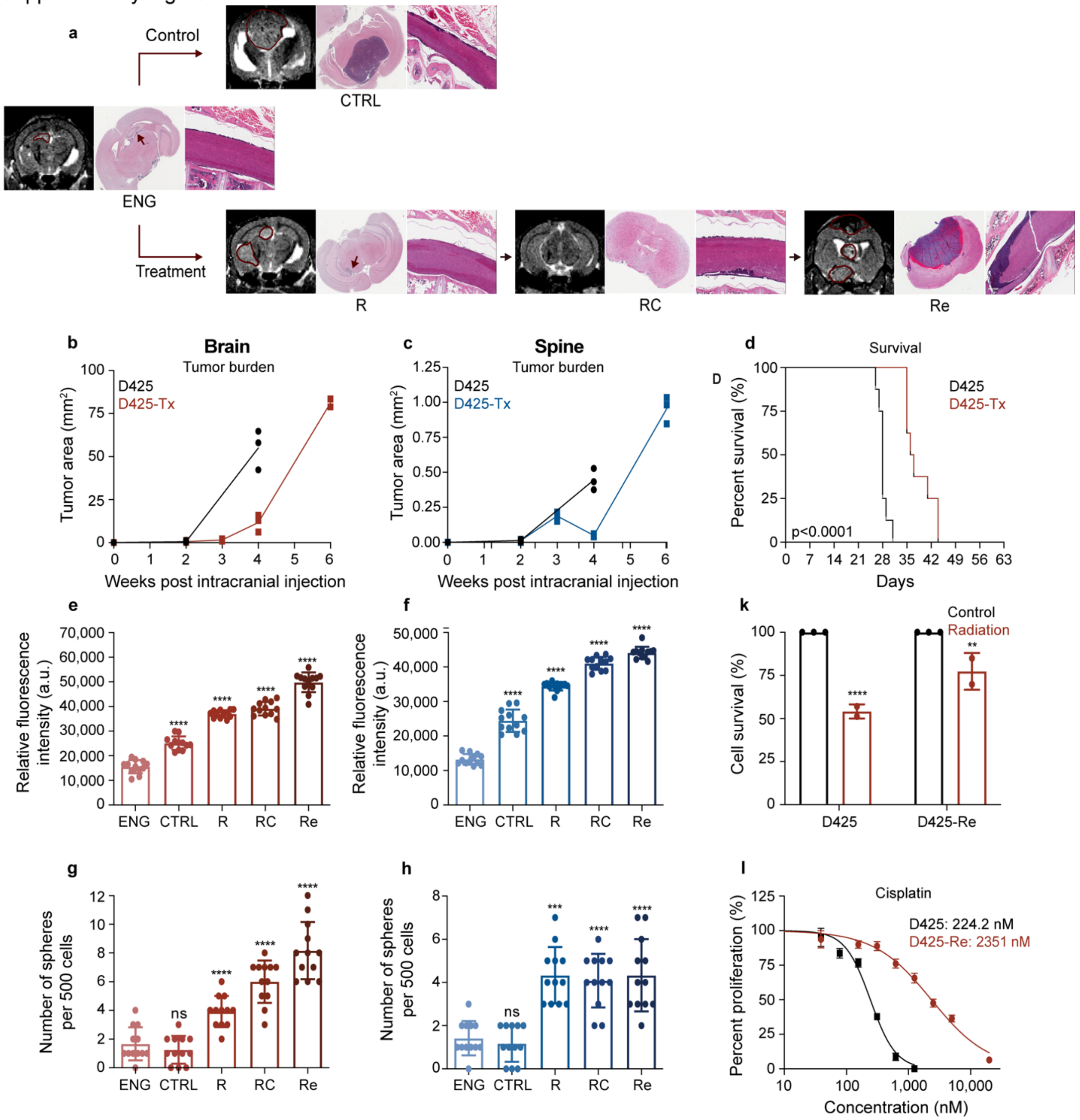

i

Sample		Frequency of self-renewing cells
	D425	1 in 219 cells
	ENG	1 in 166 cells
	CTRL	1 in 149 cells
	R	1 in 87 cells
	RC	1 in 72 cells
	Re	1 in 56 cells

j

Sample		Frequency of self-renewing cells
	ENG	1 in 157 cells
	CTRL	1 in 156 cells
	R	1 in 141 cells
	RC	1 in 122 cells
	Re	1 in 111 cells

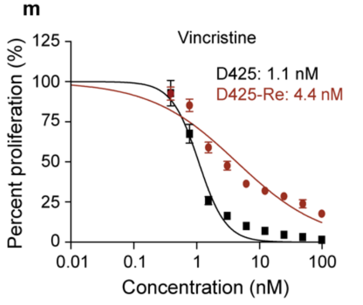

Figure S1: Functional profiling of D425 cells through *in vivo* chemoradiotherapy. (a) Schematic representation of the novel PDX mouse-adapted therapy model using patient derived human G3 MB. Brains and spines collected at engraftment (ENG), post radiation (R), post radiation and chemotherapy (RC) and at relapse (Re) were processed and stained with H&E. Representative MRI images showing brain tumor burden in mice undergoing *in vivo* chemoradiotherapy. Quantified changes in tumor burden in **(b)** brains and **(c)** spines of xenografted mice through therapy (n=3/timepoint). **(d)** Kaplan-Meier curve demonstrating survival benefit of mice undergoing *in vivo* chemoradiotherapy (n=8/treatment arm). Proliferation assay on cells isolated from **(e)** brains and **(f)** spines of mice undergoing *in vivo* chemoradiotherapy (n=3/timepoint). Changes in self-renewing potential of cells isolated from **(g)** brains and **(h)** spines of mice undergoing *in vivo* chemoradiotherapy (n=3/timepoint). Fraction of self-renewing cells in cultures derived from **(i)** brains and **(j)** spines of mice undergoing *in vivo* chemoradiotherapy (n=3/timepoint). Changes in sensitivity of recurrent D425 cells to *in vitro* **(k)** radiation, **(l)** cisplatin and **(m) v**incristine treatment. Bars represent mean of at least three technical replicates. *$p \leq 0.05$, **$p \leq 0.001$, ***$p \leq 0.0001$; ****$p \leq 0.00001$; unpaired t-test or one-way ANOVA with Sidak's method for multiple comparisons.

Supplementary Figure 2

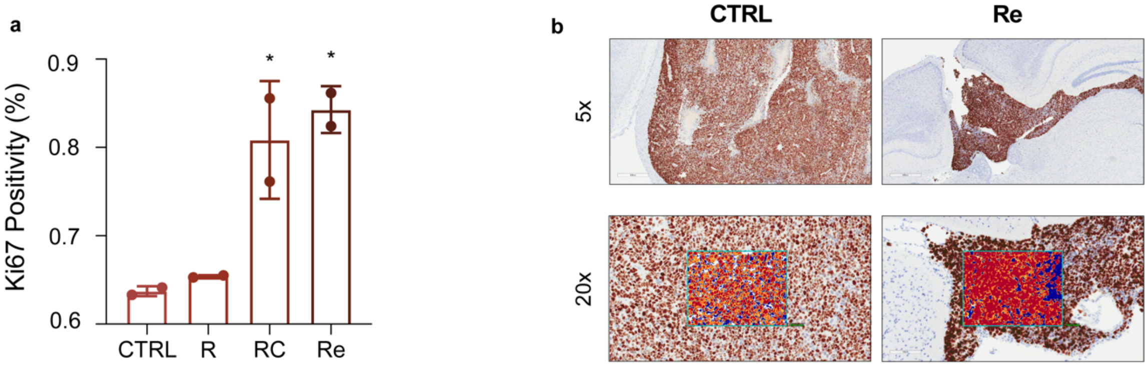

Figure S2: Ki-67 staining of brains collected through therapy. (a). Quantified Ki-67 staining positivity of brain samples collected at predetermined timepoints. (b). Representative images of Ki-67 staining of the collected brain samples. Bars represent mean of two technical replicates. *$p \leq 0.05$; unpaired t-test or one-way ANOVA with Sidak's method for multiple comparisons.

Supplementary Figure 3

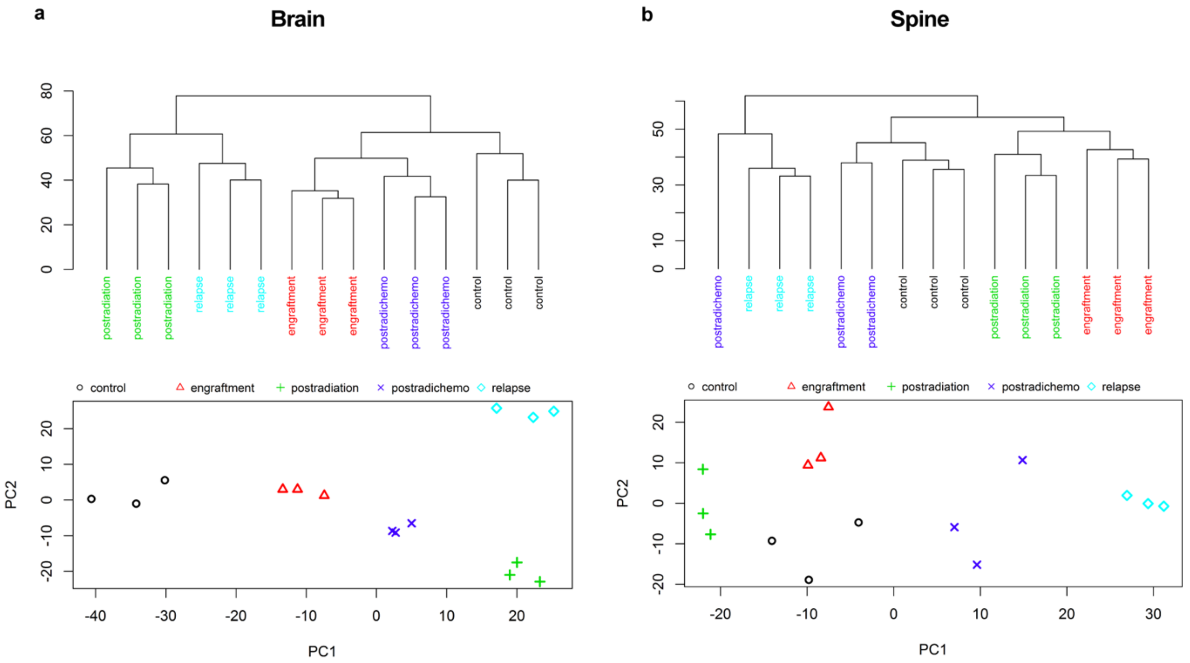

Figure S3: Technical validation and pathway analysis of RNA-seq data. Principal component analysis (PCA) of HD-MB03 cells collected from **(a)** brains and **(b)** spines though *in vivo* chemoradiotherapy.

Supplementary Figure 4

a

Brain

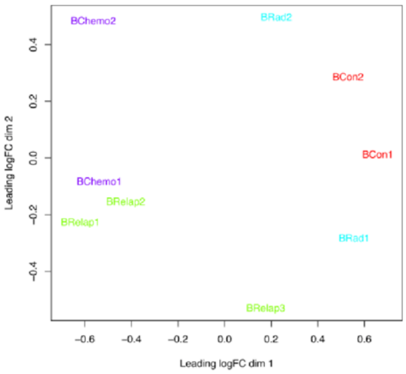

	Post-radiation		Post-chemotherapy		Relapse	
	Gene	logFC	Gene	logFC	Gene	logFC
Up-regulated genes	BPIFB4	1.10	BPIFB4	2.77	BPIFB4	2.67
	ID3	1.06	ID3	2.67	ID1	2.17
	ID1	0.96	ID1	2.28	HIST2H2AA4	1.46
	ID2	0.72	ID2	1.95	SAMD11	1.43
	NOL6	0.71	CYR61	1.82	PROM1	1.27
	SAMD11	0.67	CLDN1	1.46	BOLA1	1.20
	CRABP2	0.59	EFEMP1	1.46	H2AFJ	1.12
	PROM1	0.59	AEN	1.46	TIMM23	1.05
	MAP2K2	0.58	SERPINE2	1.40	NSUN2	1.05
	CAMK1D	0.57	C2orf80	1.28	PSMB10	1.03
Down-regulated genes	BEX1	-0.75	CACNA1G	-1.40	RPS4XP21	-1.20
	PFKFB4	-0.77	CDK5R1	-1.40	MFAP3L	-1.20
	RPL36AP39	-0.77	PPM1M	-1.43	SPTBN1	-1.27
	RPL7P8	-0.77	PRDM1	-1.44	RP11-83J16.1	-1.27
	RPL7P59	-0.78	SNORD3D	-1.48	RP11-745A24.1	-1.28
	RCVRN	-0.80	LINC00599	-1.51	KATNBL1	-1.37
	SCG3	-1.04	ERN1	-1.59	TAF1D	-1.40
	RPL7P24	-1.23	HSP90AA2P	-1.70	SNORA70	-1.44
	PRND	-1.47	SGIP1	-1.71	PDE6H	-1.55
	GDF15	-10.73	PRPH2	-1.77	RPL36AP39	-1.83

b

Spine

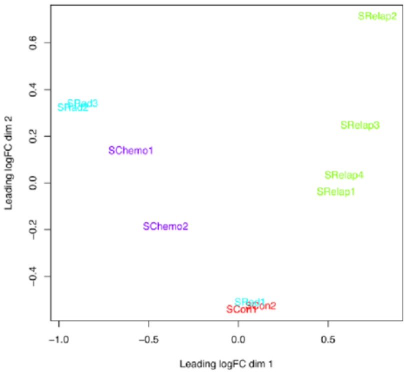

	Post-radiation		Post-chemotherapy		Relapse	
	Gene	logFC	Gene	logFC	Gene	logFC
Up-regulated genes	BCO1	1.97	BPIFB4	2.36	TEX19	2.56
	KRT18P31	1.82	ID3	1.99	HIST1H2BD	2.03
	KRT18P55	1.81	ID1	1.83	HIST1H2BE	1.98
	GSTA2	1.62	PRRX2	1.71	ALDOC	1.78
	NEFH	1.52	BMP7	1.59	BEX2	1.74
	CTSV	1.48	IGFBP3	1.58	HELZ2	1.62
	KRT18P13	1.45	TAGLN	1.57	KLF11	1.56
	KCNF1	1.43	KCNF1	1.55	RAB3IL1	1.55
	ID2	1.36	ACTA2	1.46	BEX4	1.53
	ACAT2	1.36	KRT18P55	1.43	CEBPB	1.50
Down-regulated genes	PRDM1	-1.68	INSM2	-1.40	HIBCH	-1.35
	SYP	-1.71	BRSK1	-1.40	LARP6	-1.41
	GNGT1	-1.73	FCAR	-1.42	CTXN1	-1.44
	RAB26	-1.88	SSTR2	-1.43	CTC-512J14.7	-1.44
	COX15	-1.89	ZNF483	-1.44	ST6GALNAC5	-1.53
	INSM2	-2.18	SHROOM4	-1.46	HNRNPA1P35	-1.58
	PDC	-2.21	XRCC2	-1.53	SYP	-1.60
	IFI44	-2.58	POU4F2	-1.54	FAM19A4	-1.65
	PRND	-2.60	SEMA3E	-1.54	INSM2	-2.06
	FAM57B	-2.92	FAM19A4	-1.59	TFPI2	-2.45

Figure S4: Differential gene expression analysis generated from gene microarray. PCA and logFC values of top 10 up- and down- regulated genes in D425 cells collected from **(a)** brain and **(b)** spines through therapy.

Supplementary Figure 5

a

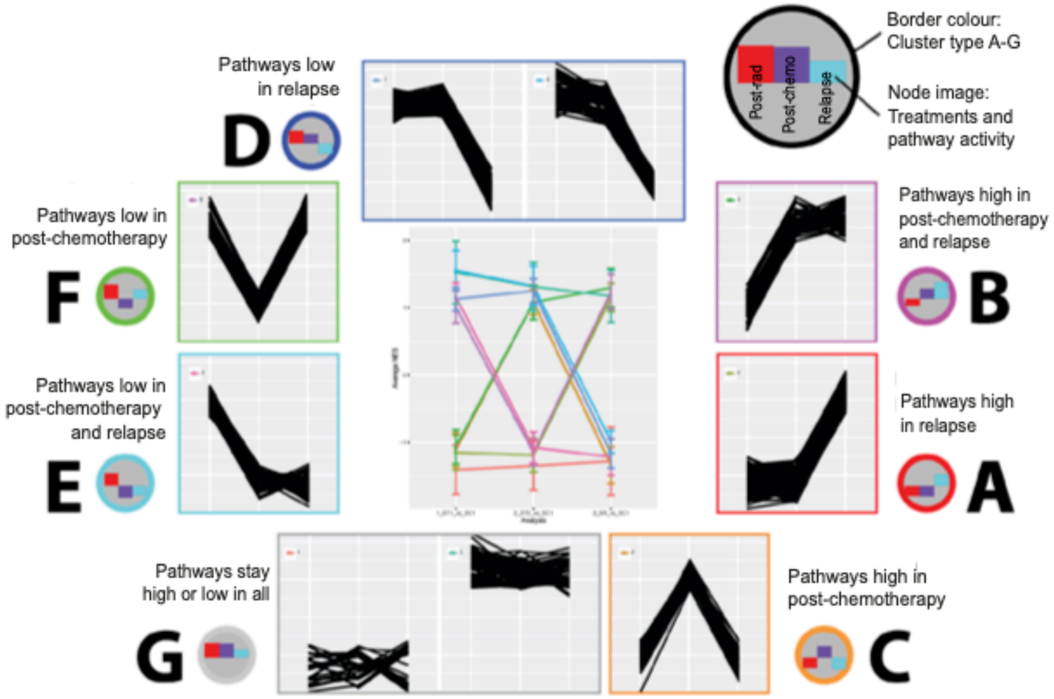

b

Cluster type	Brain		Spine	
	Number	Percentage (%)	Number	Percentage (%)
A	25	6.30	194	22.80
B	219	55.16	65	7.64
C	18	4.53	57	6.70
D	20	5.04	305	35.84
E	37	9.32	55	6.46
F	43	10.83	65	7.64
G	35	8.82	110	12.93

c

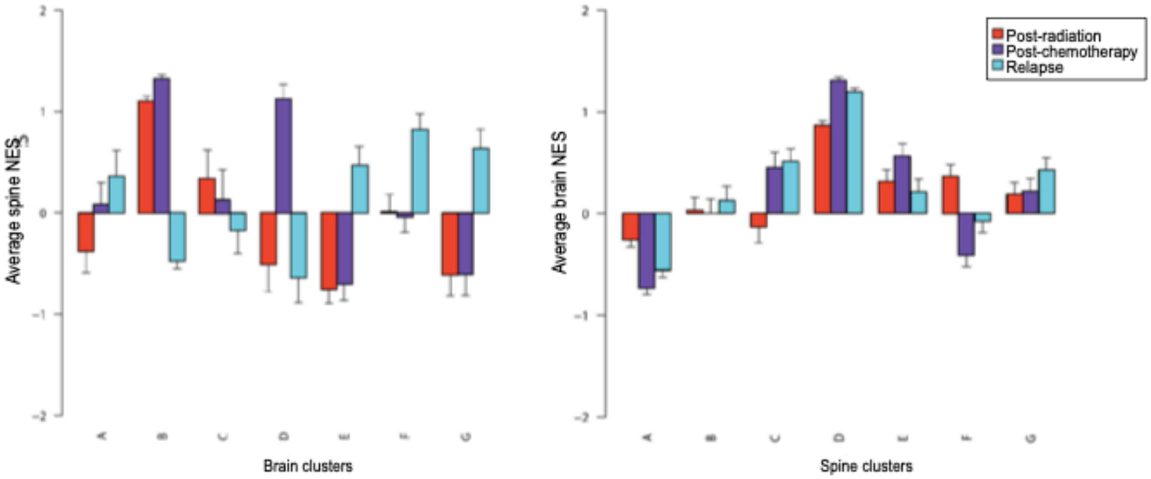

Figure S5: Separation of pathways by pattern of change. (**a, b**) Clustering of pathways activated after radiation, chemotherapy and at relapse; pathways with z-value differences greater than 2.33 ($p < 0.01$) were used for clustering. **(c)** Distinct patterns of change in pathway clusters identified in brain and spine samples.

Supplementary Figure 6

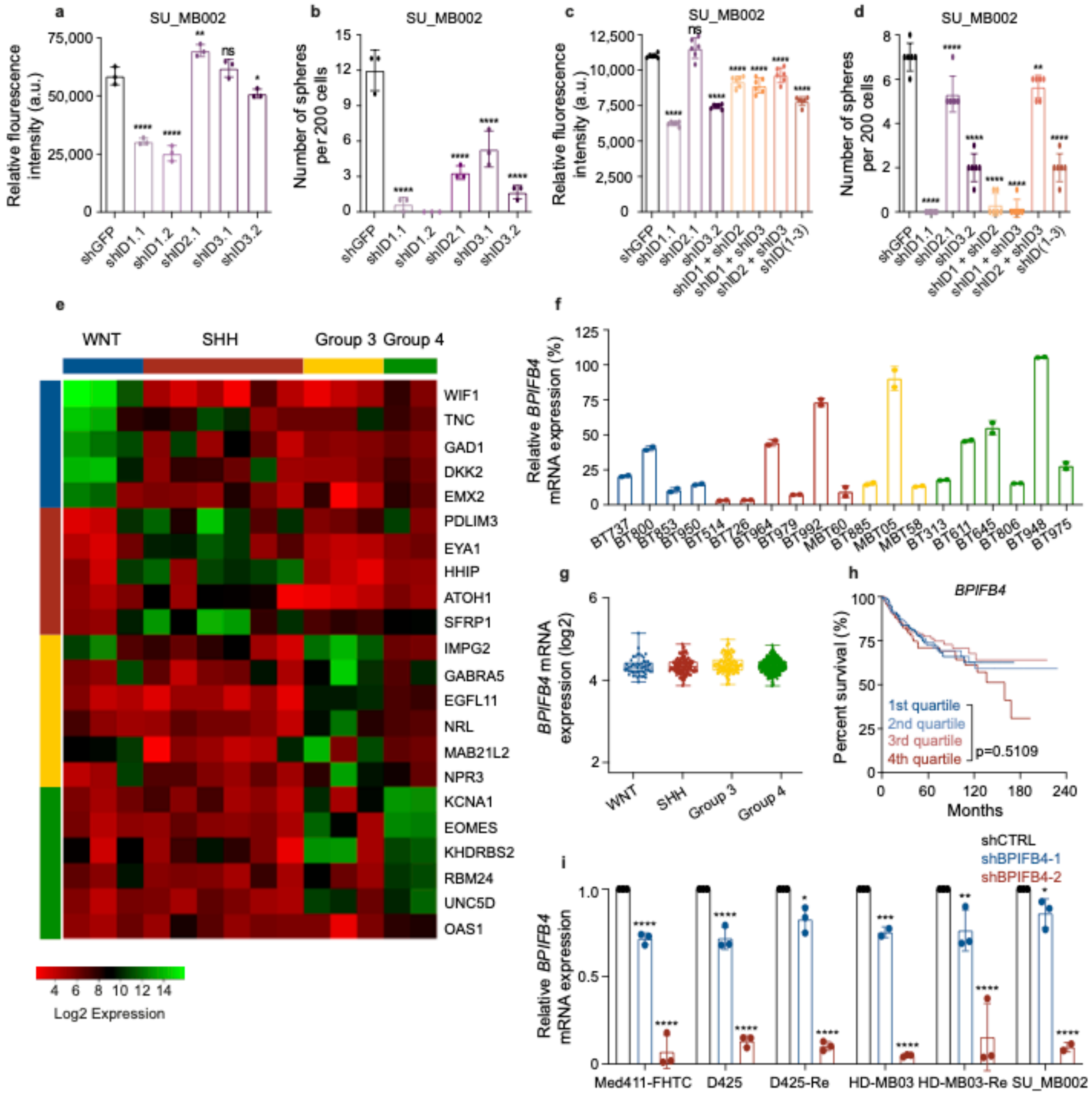

Figure S6: Validation of genes identified from comparative gene expression profiling of samples isolated through *in vitro* chemoradiotherapy. Changes in **(a)** proliferation and **(b)** self-renewal in SU_MB002 post lentivector mediated KD of ID1, ID2 and ID3 in SU_MB002 alone and in **(c, d)** combinations. **(e)** Subgroup affiliation of MB patient samples based on expression of subgroup specific genes on NanoString nCounter platform. **(g)** Relative mRNA expression of *BPIFB4* across 19 patient MB tissues. **(g)** mRNA expression of *BPIFB4* across 628 MB samples described in Cavalli *et al.*(*5*) **(h)** Kaplan-Meir curve comparing overall survival in MB patients (n=628) based on *BPIFB4* mRNA expression levels. **(i)** Validation of BPIFB4 KD across five MB cell lines. Bars represent mean of at least three technical replicates. *$p \leq 0.05$, **$p \leq 0.001$, ***$p \leq 0.0001$; ****$p \leq 0.00001$; unpaired t-test or one-way ANOVA with Sidak's method for multiple comparisons.

Supplementary Figure 7

a

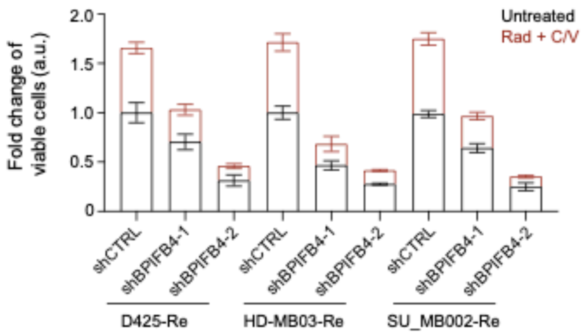

b

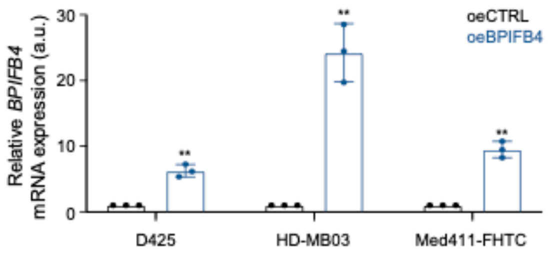

c

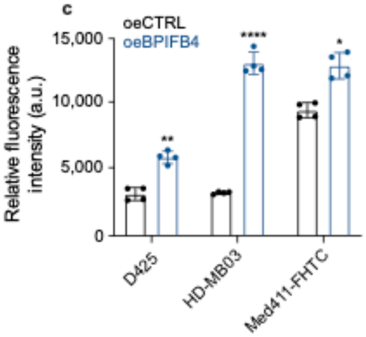

d

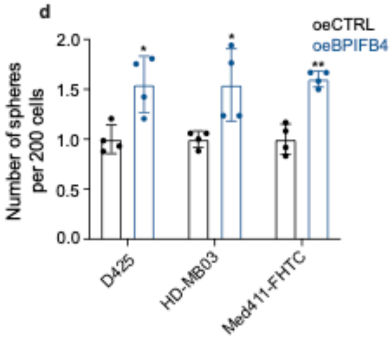

e

	oeCTRL	oeBPIFB4
D425	1 in 139 cells	1 in 43 cells
HD-MB03	1 in 117 cells	1 in 58 cells
Med411-FHTC	1 in 162 cells	1 in 127 cells

f

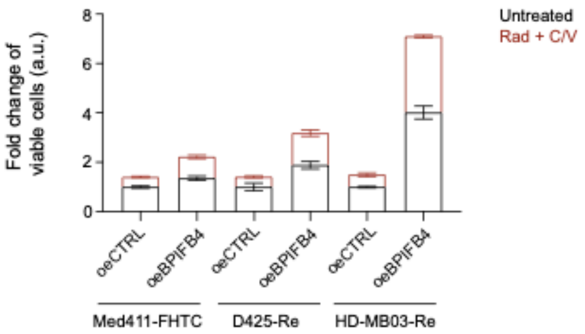

Figure S7: Modulation of BPIFB4 levels affects sensitivity to *in vitro* chemoradiotherapy in recurrent G3 MBs and functional phenotype of primary G3 MB. (a) shRNA mediated KD of BPIFB4 lead to an increased sensitivity of recurrent G3 MB cells to combined *in vitro* treatment of radiation, cisplatin and vincristine. All treatments were delivered at pre-determined IC50 values. **(b)** Validation of increased BPIFB4 mRNA levels post lentiviral transduction with overexpression vectors. Changes in **(c)** proliferation, **(d)** sphere forming potential and **(e)** frequency of self-renewing cells in primary G3 MB cells post BPIFB4 overexpression. **(f)** Exogenous overexpression of BPIFB4 reduces sensitivity of primary G3 MB cells to *in vitro* treatment of radiation, cisplatin and vincristine. Bars represent mean of at least three technical replicates. *$p \leq 0.05$, **$p \leq 0.001$, ***$p \leq 0.0001$; ****$p \leq 0.00001$; unpaired t-test or one-way ANOVA with Sidak's method for multiple comparisons.

Supplementary Figure 8

a

Array coordinates	1 2	3 4	5 6	7 8	9 10	11 12	13 14	15 16	17 18
**A**	Reference spot	p38a (T180/Y182)	ERK 1/2 (T202/Y204, T221/Y223)	JNK 1/2/3 (T183/Y185, T221/Y223)	GSK-3a/b (S21/59)	N/A	p53 (S392)	N/A	Reference spot
**B**	N/A	EGFR (Y1086)	MSK 1/2 (S376/S360)	AMPKa1 (T183)	Akt1/2/3 (S473)	Akt1/2/3 (T308)	p53 (S46)	N/A	N/A
**C**	TOR (S2448)	CREB (S133)	HSP27 (S78/S82)	AMPKa2 (T172)	B-Catenin	p70 26 Kinase (T389)	p53 (S15)	c-Jun (S63)	N/A
**D**	Src (Y419)	Lyn (Y397)	Lck (Y394)	STAT2 (Y689)	STAT5a (Y694)	p70 26 Kinase (T421/S424)	RSK 1/2/3 (S380/S386/ S377)	eNOS (S1177)	N/A
**E**	Fyn (Y420)	Yes (Y426)	Fgr (Y412)	STAT6 (Y641)	STAT5b (Y699)	STAT3 (Y705)	P27 (T198)	PLC-γ1 (Y783)	N/A
**F**	Hck (Y411)	Chk-2 (T68)	FAK (Y397)	PDGF-Rb (Y751)	STAT5a/b (Y694/Y699)	STAT3 (S727)	WNK1 (T60)	PYK2 (Y402)	N/A
**G**	Reference spot	PRAS40 (T246)	N/A	N/A	PBS (negative control)	HSP60 (T421/S424)	N/A	N/A	PBS (negative control)

b

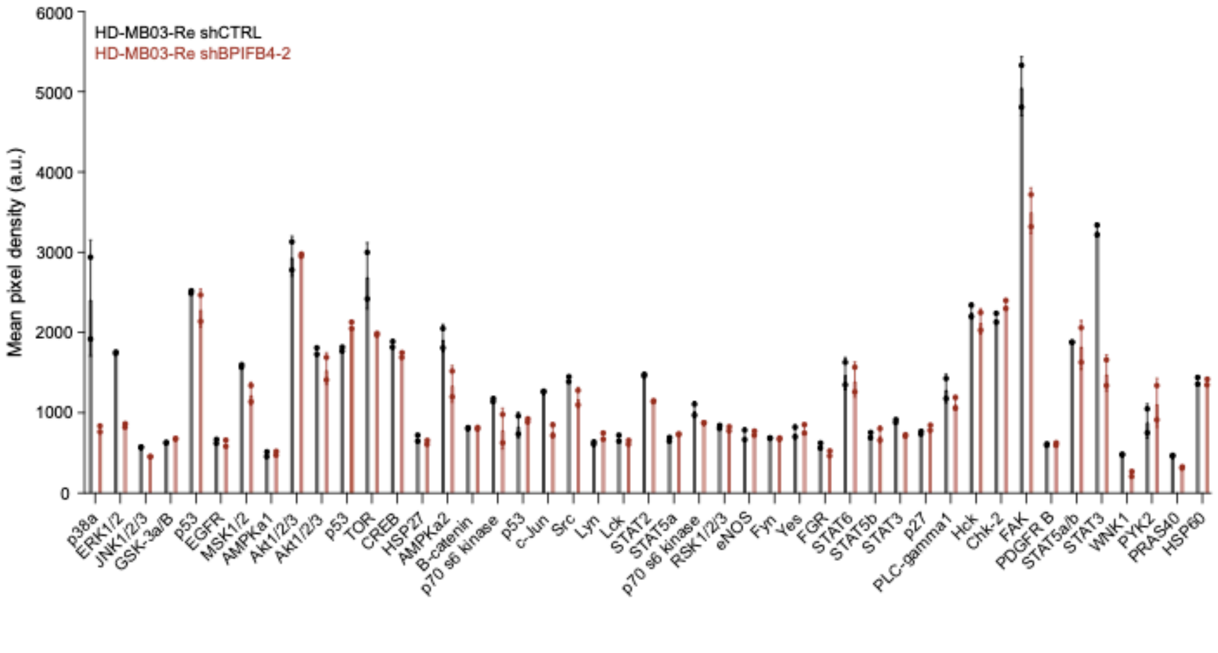

Figure S8: Quantitative analysis of Human Phospho-Kinase Array. (a) Membrane layout. Membrane 1 includes rows A through G and columns 1 to 10, while Membrane 2 includes rows A through G and columns 11 to 18. **(b)** Quantification of the array results. While the bar graph summarizes changes across all probes measured at different exposures, the single target changes were measured at a specific exposure.

Supplementary Figure 9

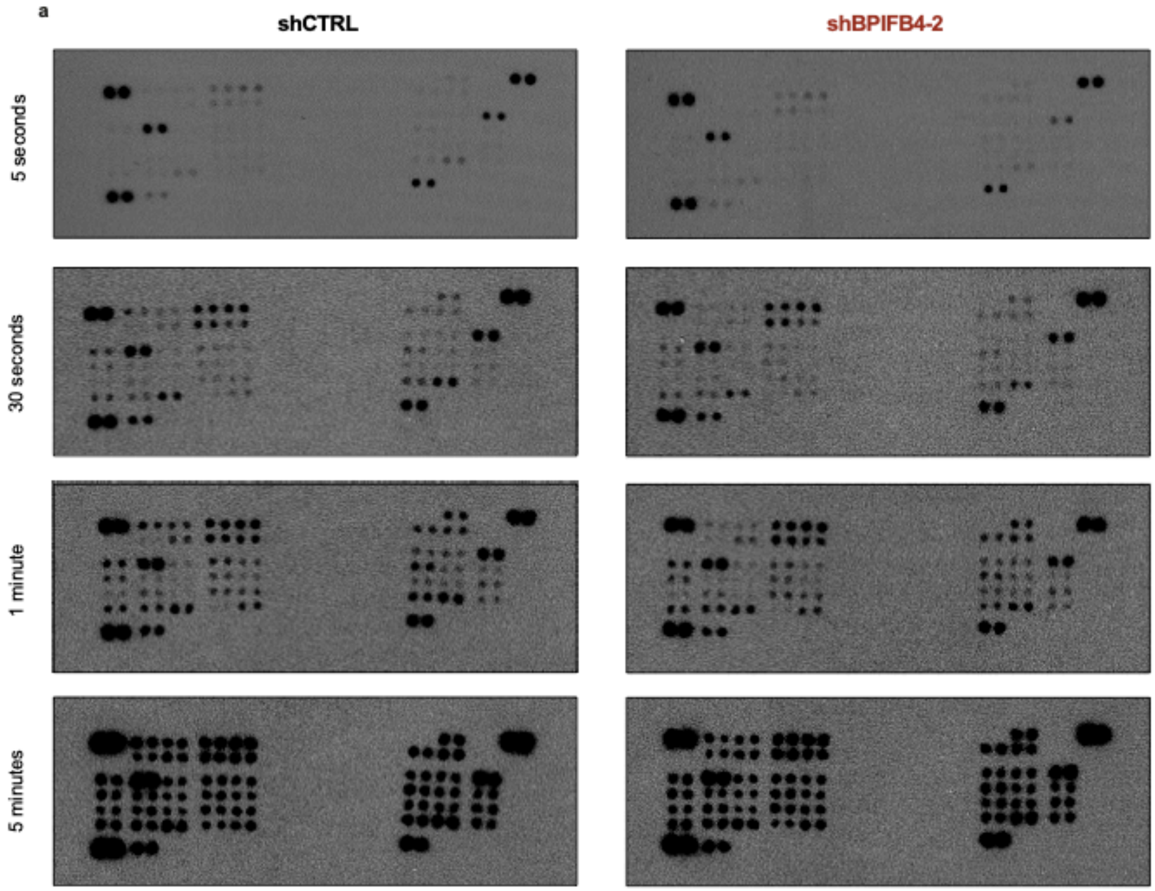

Figure S9: Full uncut results of Human Phospho-Kinase array. The array was used as per manufacture's guidelines with no modifications to the protocol. Several exposures were imaged to allow for proper visualization of all targets.

Supplementary Figure 10

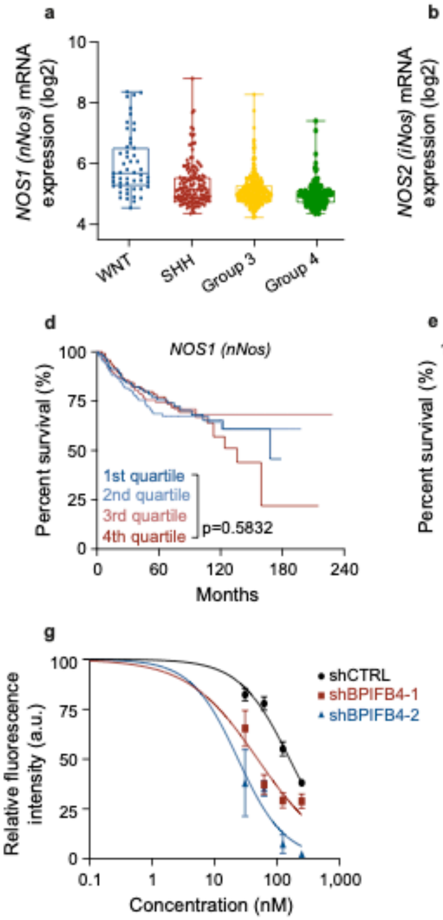

Figure S10: Comparison of nitric oxide synthases expression and prognostic value across MB samples. mRNA expression of **(a)** *NOS1* **(b)** *NOS2* and **(c)** *NOS3* across MB samples (n=628) described in Cavalli *et al*.(*5*) Kaplan-Meir curve comparing overall survival in MB patients (n=628) based on **(d)** *NOS1* **(e)** *NOS2* and **(f)** *NOS3* mRNA expression levels. **(g)** Increased sensitivity of HD-MB03-Re MB cells to DPI treatment after transduction with BPIFB4 KD lentivector.

Table S1: Differential gene expression analysis generated from RNA-seq profiling. LogFC values of top 20 up- and down- regulated genes in HD-MB03 cells isolated from brains and spines of mice undergoing *in vivo* chemoradiotherapy.

Supplementary Table 1

Brain

	Gene	Post-rad vs. Engraft	Post-chemo vs. Engraft	Relapse vs. Engraft
Up-regulated genes	BPIFB4	9.76	6.90	3.27
	CHRNB4	5.17	3.96	4.34
	THEM5	5.72	3.73	2.34
	DLX2	4.09	3.63	3.17
	MT3	4.78	2.47	2.35
	LRRC17	3.87	2.47	2.28
	SLC44A5	3.69	2.14	2.37
	ARHGEF4	1.63	3.04	3.45
	IL6R	3.45	2.87	1.73
	SOX8	3.13	1.65	3.21
	FEZF1-AS1	2.35	1.74	3.76
	INHBB	3.49	2.14	1.97
	EPHB1	3.70	2.51	1.24
	SEMA6B	2.67	2.07	2.51
	SLC8A3	2.77	1.86	2.53
	RIPPLY2	2.41	1.70	2.59
	SNTB1	2.07	2.00	2.60
	TMEM200A	3.01	2.18	1.38
	DLX1	2.58	2.20	1.67
	DRAXIN	2.56	1.74	2.11
Down-regulated genes	ACTC1	-3.41	-9.80	-7.48
	CCDC136	-2.86	-3.43	-3.23
	CACNA1H	-4.05	-2.75	-2.58
	KLHDC8A	-2.91	-2.17	-3.02
	VWA5B1	-3.17	-1.47	-2.81
	CCDC175	-2.05	-1.92	-2.66
	KLHL32	-2.25	-1.37	-2.01
	RP11-413P11.1	-0.98	-1.44	-2.89
	KCNV2	-2.38	-1.46	-1.46
	ALPL	-2.03	-1.44	-1.83
	DOC2B	-2.65	-1.31	-1.30
	SYT2	-1.79	-1.47	-1.87
	ANO9	-1.76	-1.46	-1.81
	PAPLN	-2.09	-1.66	-1.19
	GREB1	-1.44	-1.11	-2.32
	GYLTL1B	-2.84	-0.60	-1.40
	SRL	-2.37	-1.12	-1.25
	NMU	-1.24	-1.42	-2.06
	FRMD3	-2.07	-1.56	-1.02
	SALL1	-1.95	-1.63	-1.02

Spine

	Gene	Post-rad vs. Engraft	Post-chemo vs. Engraft	Relapse vs. Engraft
Up-regulated genes	SRL	0.91	3.11	5.46
	CREG2	3.47	3.16	2.79
	HBE1	2.07	1.62	2.65
	C2orf82	1.48	2.06	2.20
	RINL	1.35	1.21	1.64
	ARHGAP27	1.56	1.04	1.24
	NEURL1B	0.93	0.88	1.49
	SLC25A45	1.11	1.01	1.17
	TNNC2	0.62	0.59	2.07
	THNSL2	1.16	0.96	1.06
	ALPK2	0.83	1.01	1.31
	CDHR1	1.30	0.62	1.16
	IQCH-AS1	0.92	0.88	1.24
	FABP3	0.78	0.98	1.22
	RTBDN	0.89	1.20	0.88
	SNTB1	0.87	0.79	1.12
	FAM179A	0.87	0.70	1.19
	DDIT4L	0.78	0.54	1.32
	SPOCK1	0.60	0.66	1.34
	MACROD1	0.57	0.94	1.10
Down-regulated genes	RP11-555J4.4	-1.47	-1.20	-1.45
	LINC00085	-0.61	-0.80	-1.38
	ADRA2C	-0.86	-0.58	-1.32
	TPK1	-0.70	-0.50	-1.08
	ARHGAP6	-0.74	-0.64	-0.88
	PLAGL1	-0.41	-0.89	-0.83
	ARHGEF18	-0.59	-0.51	-0.71
	HSPA5	-0.34	-0.60	-0.86
	SHOX2	-0.61	-0.52	-0.67
	RP5-991G20.1	-0.56	-0.45	-0.73
	CDH2	-0.50	-0.51	-0.73
	TLN2	-0.29	-0.48	-0.82
	DNAJB1	-0.46	-0.81	-0.32
	PKD1P6	-0.46	-0.35	-0.55
	RP11-395G23.3	-0.53	-0.41	-0.40
	NBPF1	-0.46	-0.36	-0.42
	MROH6	-0.29	-0.58	-0.35
	DNAJC3	-0.28	-0.45	-0.47
	HERPUD1	-0.30	-0.29	-0.59
	PLCB4	-0.38	-0.36	-0.40

Table S2: Clusters of top 4 pathways (by p-value) with NES in the D425 brain samples.

Supplementary Table 2

Pathways in brain	Cluster	Post-rad	Post-chemo	Relapse	p_diff
ACTIVATION OF RRNA EXPRESSION BY ERCC6 (CSB) AND EHMT2 (G9A)-REACTOME	A	-0.84	-0.67	2.35	1.33E-04
HDMS DEMETHYLATE HISTONES-REACTOME	A	-0.72	-1.14	1.60	1.63E-04
NONHOMOLOGOUS END-JOINING (NHEJ)-REACTOME	A	-0.89	-0.93	1.73	2.04E-04
POSITIVE EPIGENETIC REGULATION OF RRNA EXPRESSION-REACTOME	A	-0.76	-0.83	2.03	2.15E-04
PROTEIN-DNA COMPLEX SUBUNIT ORGANIZATION-GOBP	B	-0.91	1.79	2.00	1.10E-04
PROGRAMMED CELL DEATH-GOBP	B	-0.97	1.32	1.02	1.18E-04
REGULATION OF GENE EXPRESSION, EPIGENETIC-GOBP	B	-0.90	1.11	1.69	1.23E-04
NUCLEOSOME ASSEMBLY-GOBP	B	-0.87	1.39	1.92	1.29E-04
NEGATIVE REGULATION OF CELLULAR COMPONENT MOVEMENT-GOBP	C	-1.20	1.17	-0.99	1.13E-03
NEGATIVE REGULATION OF CELL MORPHOGENESIS INVOLVED IN DIFFERENTIATION-GOBP	C	-0.84	1.32	-1.08	1.94E-03
PIGMENTATION-GOBP	C	-1.57	0.83	-1.03	2.18E-03
INTRASPECIES INTERACTION BETWEEN ORGANISMS-GOBP	C	-1.38	1.14	-1.18	2.89E-03
SELENOAMINO ACID METABOLISM-REACTOME	D	1.71	1.20	-0.85	4.19E-04
EUKARYOTIC TRANSLATION ELONGATION-REACTOME	D	1.28	1.07	-1.23	5.12E-04
PROTEIN TARGETING TO MEMBRANE-GOBP	D	1.16	1.25	-1.17	5.29E-04
P73 TRANSCRIPTION FACTOR NETWORK-NCI-NATURE CURATED DATA	D	1.09	1.48	-0.97	1.29E-03
POSITIVE REGULATION OF AXON EXTENSION-GOBP	E	0.96	-1.83	-1.58	1.42E-04
UPTAKE AND ACTIONS OF BACTERIAL TOXINS-REACTOME	E	1.02	-1.62	-0.77	4.42E-04
POSITIVE REGULATION OF DEVELOPMENTAL GROWTH-GOBP	E	0.89	-1.68	-1.17	5.52E-04
GABA-B_RECEPTOR_II_SIGNALING-PANTHER PATHWAY	E	1.10	-1.51	-0.87	7.49E-04
CELLULAR RESPONSE TO UNFOLDED PROTEIN-GOBP	F	1.55	-0.95	0.95	1.21E-04
ANTIMICROBIAL HUMORAL RESPONSE-GOBP	F	0.41	-1.07	1.77	1.76E-04
ANTIBACTERIAL HUMORAL RESPONSE-GOBP	F	0.41	-1.06	1.75	2.15E-04
POSITIVE REGULATION OF GENE EXPRESSION, EPIGENETIC-GOBP	F	0.89	-1.05	1.69	2.16E-04
ACID SECRETION-GOBP	G	-0.83	-1.96	-1.40	1.54E-03
SIGNAL RELEASE FROM SYNAPSE-GOBP	G	-0.67	-1.68	-1.23	1.65E-03
SYNAPTIC TRANSMISSION-GOBP	G	-0.91	-1.51	-1.22	1.87E-03
TRANS-SYNAPTIC SIGNALING-GOBP	G	-0.92	-1.53	-1.24	1.90E-03

Table S3: Clusters of top 4 pathways (by p-value) with NES in the D425 spine samples.

Supplementary Table 3

Pathways in spine	Cluster	Post-rad	Post-chemo	Relapse	p_diff
REGULATION OF INTERLEUKIN-4 PRODUCTION-GOBP	A	-1.24	-1.21	1.63	1.26E-04
CELLULAR RESPONSE TO CARBOHYDRATE STIMULUS-GOBP	A	-1.83	-1.38	1.02	1.36E-04
VESICLE ORGANIZATION-GOBP	A	-1.17	-1.13	1.27	1.46E-04
MUSCLE CONTRACTION-REACTOME	A	-1.32	-1.11	1.22	1.57E-04
NEGATIVE REGULATION OF CELL ACTIVATION-GOBP	B	-0.99	1.14	1.66	1.01E-04
REGULATION OF RHODOPSIN MEDIATED SIGNALING PATHWAY-GOBP	B	-1.74	1.17	0.98	1.23E-04
CELLULAR RESPONSE TO TOPOLOGICALLY INCORRECT PROTEIN-GOBP	B	-0.97	0.98	1.65	1.51E-04
ACTIVATION OF RRNA EXPRESSION BY ERCC6 (CSB) AND EHMT2 (G9A)-REACTOME	B	-0.95	1.31	1.79	1.83E-04
NUCLEAR-TRANSCRIBED MRNA CATABOLIC PROCESS, NONSENSE-MEDIATED DECAY-GOBP	C	-0.89	1.41	-1.18	1.31E-04
ASSOCIATION OF TRIC CCT WITH TARGET PROTEINS DURING BIOSYNTHESIS-REACTOME	C	-0.98	0.96	-1.78	1.55E-04
PROSTACYCLIN SIGNALLING THROUGH PROSTACYCLIN RECEPTOR-REACTOME	C	-1.30	1.13	-1.69	1.59E-04
THROMBIN SIGNALLING THROUGH PROTEINASE ACTIVATED RECEPTORS (PARS)-REACTOME	C	-1.11	1.17	-1.59	1.69E-04
TRANSCRIPTION FROM RNA POLYMERASE II PROMOTER-GOBP	D	1.51	1.35	-0.94	1.00E-04
HYDROGEN ION TRANSMEMBRANE TRANSPORT-GOBP	D	1.06	1.69	-1.13	1.01E-04
POSITIVE REGULATION OF CYTOKINESIS-GOBP	D	1.74	1.37	-1.06	1.03E-04
NUCLEUS ORGANIZATION-GOBP	D	2.11	1.41	-0.90	1.03E-04
RESPONSE TO IONIZING RADIATION-GOBP	E	1.31	-1.29	-1.08	1.44E-04
CELLULAR CARBOHYDRATE CATABOLIC PROCESS-GOBP	E	1.17	-0.99	-1.64	1.67E-04
AXON GUIDANCE-REACTOME DATABASE ID RELEASE 56	E	0.90	-1.17	-1.42	1.94E-04
G2 DNA DAMAGE CHECKPOINT-GOBP	E	1.31	-1.42	-1.10	2.41E-04
LEARNING-GOBP	F	0.69	-1.52	1.27	1.42E-04
CYTOSKELETON ORGANIZATION-GOBP	F	1.47	-0.92	1.00	2.23E-04
ACYLGLYCEROL BIOSYNTHETIC PROCESS-GOBP	F	0.92	-1.52	1.26	2.44E-04
PLC-GAMMA1 SIGNALLING-REACTOME	F	1.11	-1.11	1.63	2.78E-04
FATTY ACID METABOLIC PROCESS-GOBP	G	0.89	0.76	1.61	6.42E-04
COPI-DEPENDENT GOLGI-TO-ER RETROGRADE TRAFFIC-REACTOME	G	1.75	1.07	0.76	6.92E-04
MICROTUBULE-BASED PROCESS-GOBP	G	1.91	0.94	0.86	7.96E-04
NEGATIVE REGULATION OF CATALYTIC ACTIVITY-GOBP	G	1.51	1.46	0.91	8.68E-04

Table S4: Demographic and clinical data of 19 MB samples curated at McMaster Children's Hospital. M – Male, F – Female, OS – Overall survival, PFS – progression free survival.

Supplementary Table 4

Subgroup	Sample	Age at diagnosis	Sex	Primary/Recurent	Pathology variant	OS (years)	PFS (years)	Status
WNT	BT737	6Y	M	Primary	N/A	3.5	3	Alive
	BT800	11Y	F	Primary	Desmoplastic	3.25	2.5	Alive
	BT853	5Y 6M	F	Primary	Anaplastic	3.25	2.5	Alive
	BT950	8Y	M	Primary	Anaplastic	2	1.5	Alive
SHH	BT514	8Y	F	Primary	Anaplastic	5.5	2	Alive
	BT726	1Y 11M	M	Primary	Desmoplastic	4	1	Alive
	BT964	11Y	F	Recurrent of BT514	Classic	2	1.5	Alive
	BT979	4M 25D	M	Primary	Anaplastic	0.25	0.17	Dead
	BT992	6M 5D	M	Recurrent of BT979	Anaplastic	0.25	0.17	Dead
	MBT60	5Y 2M	M	Recurrent of BT726	Desmoplastic	4	3	Alive
Group 3	BT885	2Y 4M	F	Primary	Desmoplastic	2.75	2	Alive
	MBT05	10M	F	Primary	Desmoplastic	1.33	0.83	Alive
	MBT58	3Y 11M	M	Primary	Desmoplastic	0.67	0.17	Alive
Group 4	BT313	16Y	M	Primary	Desmoplastic	4.5	2.17	Dead
	BT611	18Y	M	Recurrent of BT313	Desmoplastic	4.75	2.6	Dead
	BT645	10M	M	Primary	Desmoplastic	4.75	4	Alive
	BT806	3Y 11M/M	M	Primary	Desmoplastic	3.25	2.5	Alive
	BT948	10Y	M	Primary	Classic	2	1.5	Alive
	BT975	5Y 4M	M	Recurrent of BT806	Classic	3.25	1.6	Alive
